# Understanding the differences of the ligand binding/unbinding pathways between phosphorylated and non-phosphorylated ARH1 using molecular dynamics simulations

**DOI:** 10.1038/s41598-017-12031-0

**Published:** 2017-09-29

**Authors:** Jingxuan Zhu, Yishuo Lv, Xiaosong Han, Dong Xu, Weiwei Han

**Affiliations:** 1 Key Laboratory for Molecular Enzymology and Engineering of Ministry of Education, School of Life Science, Jilin University, 2699 Qianjin Street, Changchun, 130012 China; 2 Department of Electric Engineering and Computer Science, C.S. Bond Life Sciences Center, University of Missouri, Columbia, Missouri 65211 USA; 3 College of Computer Science and Technology Jilin University, 2699 Qianjin Street, Changchun, 130012 China

## Abstract

ADP-ribosylhydrolases (ARH1, ARH2 and ARH3) are a family of enzymes to catalyze ADP-ribosylation, a reversible and covalent post-translational modification (PTM). There are four phosphorylated sites (Tyr-4, Tyr-19, Tyr-20, and Tyr-205) in ARH1. To explore the structural changes and functional impact induced by phosphorylation, molecular dynamics (MD) simulations and steered molecular dynamics (SMD) simulations were performed for the phosphorylated and non-phosphorylated ARH1 with the ligands. MD simulations results indicate that: (1) Glu-25 is more frequently in the α helix group in the phosphorylated state with the adenosine-5-diphosphate-ribosylarginine (ADP-RA) complex (51.56%) than that of the non-phosphorylated state(2.12%); (2) Ser-124 and Ser-264 become less flexible in the phosphorylated state with ADP-RA complex, which helps two residues form hydrogen bonds with ADP-RA; and (3) Tyr-211 is also less flexible in the phosphorylated state with ADP-RA complex, which helps stabilize the cation-π interaction of Y211-R119. All these changes facilitate ADP-RA to bind ARH1. In addition, according to the crystal structure of adenosine-5-diphosphate-ribose (ADP-ribose) in complex with non-phosphorylated and phosphorylated ARH1, the possible unbinding pathways of ADP-ribose from non-phosphorylated and phosphorylated ARH1 were explored respectively using SMD simulations. Our results show that phosphorylated ARH1 has more ordered structures than the non-phosphorylated type.

## Introduction

ADP-ribosylation is a reversible post-translational modification (PTM) of specific target proteins (such for GTP-binding or ATP-binding proteins) and DNA^1^. The extent and duration of mono-ADP-ribosylation govern the activities of ADP-ribosylhydrolases (ARHs) and poly-ADP-ribosylglycohydrolases (PARGs), the latter of which reverse the transfer reaction by hydrolyzing the protein-ADP-ribose bonds and/or ADP-ribose–ADP-ribose bonds (see Figure S1) (*i.e*. the release of ADP-ribose from the acceptor molecule), and is catalyzed by ADP-ribosylhydrolases (ARHs) or poly-ADP-ribosylglycohydrolases (PARGs)^1–3^.

The ARH gene family comprises ARH1, ARH2, ARH3^4^ and one PARG^5^. ARH1 has been shown as an Arg-specific mono-ADP-ribosyl hydrolase that can de-ADP-ribosylate many different Arg-ADP-ribosylated target proteins^6–8^. Neither the substrate specificity nor the enzymatic activity of ARH2 is known^7^. ARH3 is indicated to transfer to de-ADP-ribosylate poly-ADP-ribosylated substrates in mitochondria and to hydrolyse *O*-acetyl-ADP-ribose, a side product of sirtuin-dependent deacetylation^7,9–11^.

Until now, no structural information is available for ADP-ribosylhydrolases. To date, only the three-dimensional structures of human and mouse ARH3^1,7,9,12^ and human ARH1 (PDB code 3HFW) have been determined.

Protein PTM by the attachment of chemical groups is used by cells from all kingdoms of life and occurs in several forms. Through the reversible covalent modification of specific amino acid side chains, enzyme activity or other protein function can be switched on and off as a rapid response to environmental stimuli. ADP-ribosylation is a PTM existing in two distinct forms, mono-and poly-ADP-ribosylation, resulting from the attachment of a single ADP-ribose moiety or a polymeric ADP-ribose chain structure, respectively, to the protein. However, the effect of the phosphorylation on ARH1 is still unknown. How do these four phosphorylated residues induce the structural changes upon the ligand binding? Do these phosphorylated residues affect the ligand binding and unbinding pathway?

Molecular dynamics (MD) simulation is a computer simulation method for studying the physical movements of macromolecules such as proteins^13^. MD has been applied mostly in chemical physics, material science and modeling of biomolecules atomic scales. These days, a new approach induces unbinding of ligands based on the ligand−receptor interactions and modeling of long-time movement of biomolecules, named steered molecular dynamics (SMD) simulations^14–16^. SMD can reveal structural changes in a protein at the atomic level for the ligand binding (unbinding) pathway through tunnel at a time scale inaccessible to standard MD. In this study, we employ MD and SMD to study structural changes induced by phosphorylation and their impact on protein binding (unbinding) pathway of ADP-ribose from ARH1 ligand-binding domain (LBD) in phosphorylated and non-phosphorylated ARH1.

The ligand binding mode and unbinding pathways of phosphorylated and non-phosphorylated ARH1 were revealed in this work, and hence provide the associated unbinding mechanism of ADP-ribose from phosphorylated and non-phosphorylated ARH1, respectively. In this case, for the two systems of ADP-RA in complex with phosphorylated and non-phosphorylated ARH1, 200 ns conventional MD simulations were applied respectively. After that, based on the 3D structure of X-ray, the possible ligand unbinding pathways were explored by using two SMD simulations, and the relevant unbinding mechanism was elucidated by analyzing the unbinding trajectories.

## Materials and Methods

### System Preparation

The starting structure of human ARH1 (the non-phosphorylated type) was taken from Protein Data Bank^17^ (PDB code 3HFW) and the phosphorylation on tyrosine was added into the phosphorylated type. The initial models of ADP-RA and ADP-ribose were downloaded from ChemSpider^18^. Geometric optimization of ADP-ribose and ADP-RA were performed at the B3LYP/6-31G* level using Gaussian09 [www.gaussian.com].

### Molecular docking

Autodock Vina^19^ was selected for docking. For the macromolecules, Kollman charges and AD4 type atoms were assigned, while hydrogen atoms were also merged into associated heavy atoms. In addition, grid box was added using AutoDock Tools program. Moreover, the docking method could reproduce the binding pose of the co-crystallized ligands in the used crystal structure.

### Conventional MD Simulations and analysis

The conventional MD simulations were performed using the NAMD software^20^ with CHARMM27 all-force field parameters^20,21^ support. For the macromolecules (human ARH1) and ligands (ADP-RA), generalized CHARMM27 all-force field parameters^20,21^ were applied. The initial models were constructed with crystallization water molecules. After that the resulting model were solvated in a cubic periodic box with TIP3P^22^ water. Counter ions (Na+, Cl^−^) neutralized the systems. The resulting models were solvated with TIP3P^22^ water in a cubic periodic boundary conditions. The distance between the periodic boundary conditions and the closest protein atom was set to 10.0 Å. Prior to the MD simulation, each system was energetically minimized through the steepest descent algorithm with 50,000 steps to avoid steric clashes or improper geometries. After the minimization, an isothermal-isobaric (NPT) simulation was run by weak coupling to a bath of constant pressure (P0 = 1 bar, coupling time = 2.0 ps). In our study, the constant temperature control was based on Langevin dynamics^23^ with a damping coefficient (gamma) of 1.0 ps. The full-system periodic electrostatics were calculated by using the particle-mesh Ewald (PME) algorithm^24^. The MD simulation was carried out for 200 ns for the two protein-ligand complexes under the normal temperature (300 K) and pressure (1 bar), using a temperature coupling time constant of 0.1 ps and a pressure coupling time constant of 2.0 ps. And three replications for two complexes have been simulated in this study (Figures S2–S3).

### Principal Component Analysis and Free Energy Landscape Analysis

Principal component analysis (PCA)^25–27^ is an effective and useful technique to reduce or simplify large and complicated movements of long trajectories generated by MD simulations. The goal of PCA is to produce a transformed set of variables, z_1,_ z_2,_…, z_p._ The indices z_i_ are called the principal components (PCs). Free Energy Landscape (FEL)^28–30^ is used to describe the energies of sets of macromolecules conformations. In detail, the first two principal components (PC1, PC2) of motions were displayed in the FEL map, projecting the trajectories on their first two principal components of motion.

### SMD Simulations

To reveal the human ARH1 processes underlying the unbinding of ADP-ribose, the center of mass of the ligands were forced to pull out it along a predefined direction using NAMD 2.10b1 version software and the CHARMM27 all-atom force field^20,21^. The direction of pulling was defined by the two points, the first point was the location of the active site, i.e. the center between the C_α_ atoms of Gly-101 and Ser-264, while another point was the centroid of phenyl ring coordinate in the ligand (ADP-ribose). Commonly implemented using a constant force or a constant velocity, SMD is useful for exploring possible permeation pathways through a macromolecule. In our study, the constant-velocity ensemble SMD simulations were performed. And the spring does not move in time accessible SMD simulation, a constant of 0.5 kcal· mol^−1^ ·A^−2^ was performed to stretch the imaginary atom from the central mass of SMD atom with constant velocity. The two ligand-receptor systems were performed in 6 ns SMD simulation. And three replications for two complexes have been simulated in this study (Figures S4–S5).

### Potential of mean force calculations

The substrate pathway can be characterized by the substrate’s free energy along it, known as the potential of mean force (PMF)^31–33^, which dictates the speed of transport and the selectivity. The adaptive biasing force (ABF)^34^ was performed to generate quasi-equilibrium trajectories from which the PMF of the NAMD software can be deduced. In our study, for the two complexes, two different reaction coordinates exist, which are the distances between the substrates’ centers of mass and Mg^2+^ toward the opening of the pathway. The reaction coordinate was subdivided into equally spaced windows, and each was simulated for 6 ns. Finally, all the separated simulations belonging to the same reaction coordinate were combined into a single PMF, while the upper and lower boundaries were changed so that they encompass the entire range.

### MM-GBSA calculations

The Molecular Mechanics/Generalized Born Surface Area (MM/GBSA) calculation^35^ was performed to calculate the binding free energies of ADP-RA with phosphorylated and non-phosphorylated ARH1, respectively. Amber 14 package was used to performed 2 ns with Amber ff99 force field parameter^36^. The binding free energy ΔG_bind_ estimated as follow:

$$\begin{array}{rcl}{{\rm{\Delta }}{\rm{G}}}_{{\rm{bind}}} & = & {{\rm{G}}}_{{\rm{complex}}}-{{\rm{G}}}_{{\rm{recptor}}}-{{\rm{G}}}_{{\rm{ligand}}}\\ {{\rm{\Delta }}{\rm{G}}}_{{\rm{bind}}} & = & {{\rm{\Delta }}{\rm{E}}}_{{\rm{gas}}}+{{\rm{\Delta }}{\rm{G}}}_{{\rm{sol}}}-{\rm{T}}{\rm{\Delta }}{\rm{S}}\\ {{\rm{\Delta }}{\rm{G}}}_{{\rm{sol}}} & = & {\rm{\Delta }}{\rm{GGB}}+{{\rm{\Delta }}{\rm{G}}}_{{\rm{SA}}}\\ {{\rm{\Delta }}{\rm{G}}}_{{\rm{SA}}} & = & {\rm{\gamma }}\cdot {\rm{\Delta }}\mathrm{SASA}\end{array}$$

where ΔE_gas_ is known as the gas-phase interaction energy, including the van der Waals and the electrostatic energy, and G_sol_ is the solvation free energy, including the polar (ΔG_GB_) and non-polar (ΔG_SA_) energies. TΔS is the change of conformational entropy, which was neglected in the present study due to the low prediction accuracy and the expensive computational cost. In this study, the atomic radii were set to the default bondi values; the GB model was calculated by GB-OBC1 and the radii were set to the mbondi values. The solute dielectric constant was set to the default (ε = 1) for both of the polar solvation energies (∆*G*_GB_), and we used 80 for the exterior dielectric constants. The LCPO method was applied and the value of γ was 0. 0072^37^.

### Data Availability

The datasets generated during and/or analysed during the current study are available from the corresponding author on reasonable request.

All data generated or analysed during this study are included in this published article (and its Supplementary Information files).

## Results and Discussion

### Conventional MD simulations

**Figure 1:**
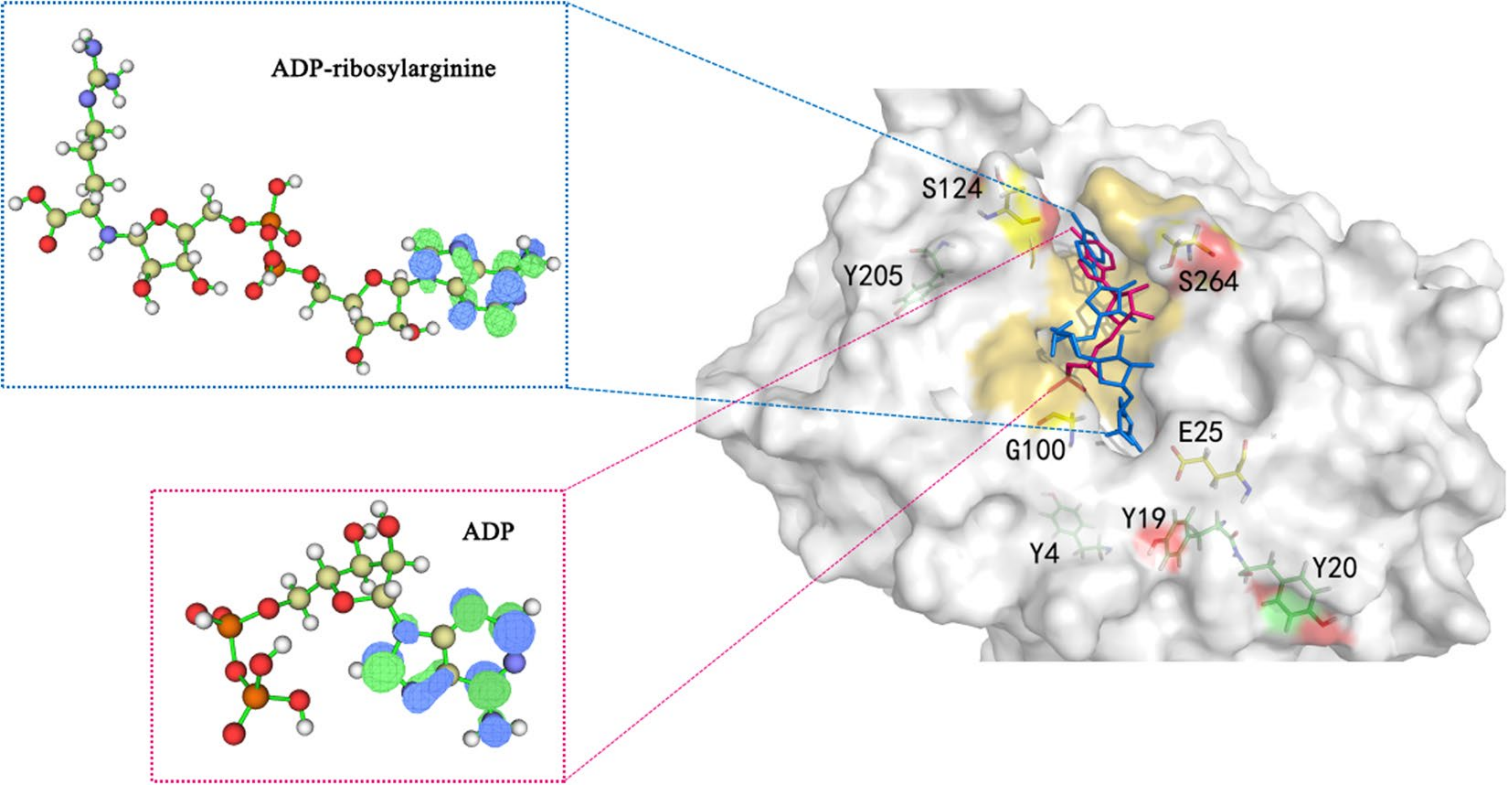
The conformation of the docked the ligand (blue) calculated by Autodock Vina and the one in the crystal structure (red) located in the active site.

**Figure 2:**
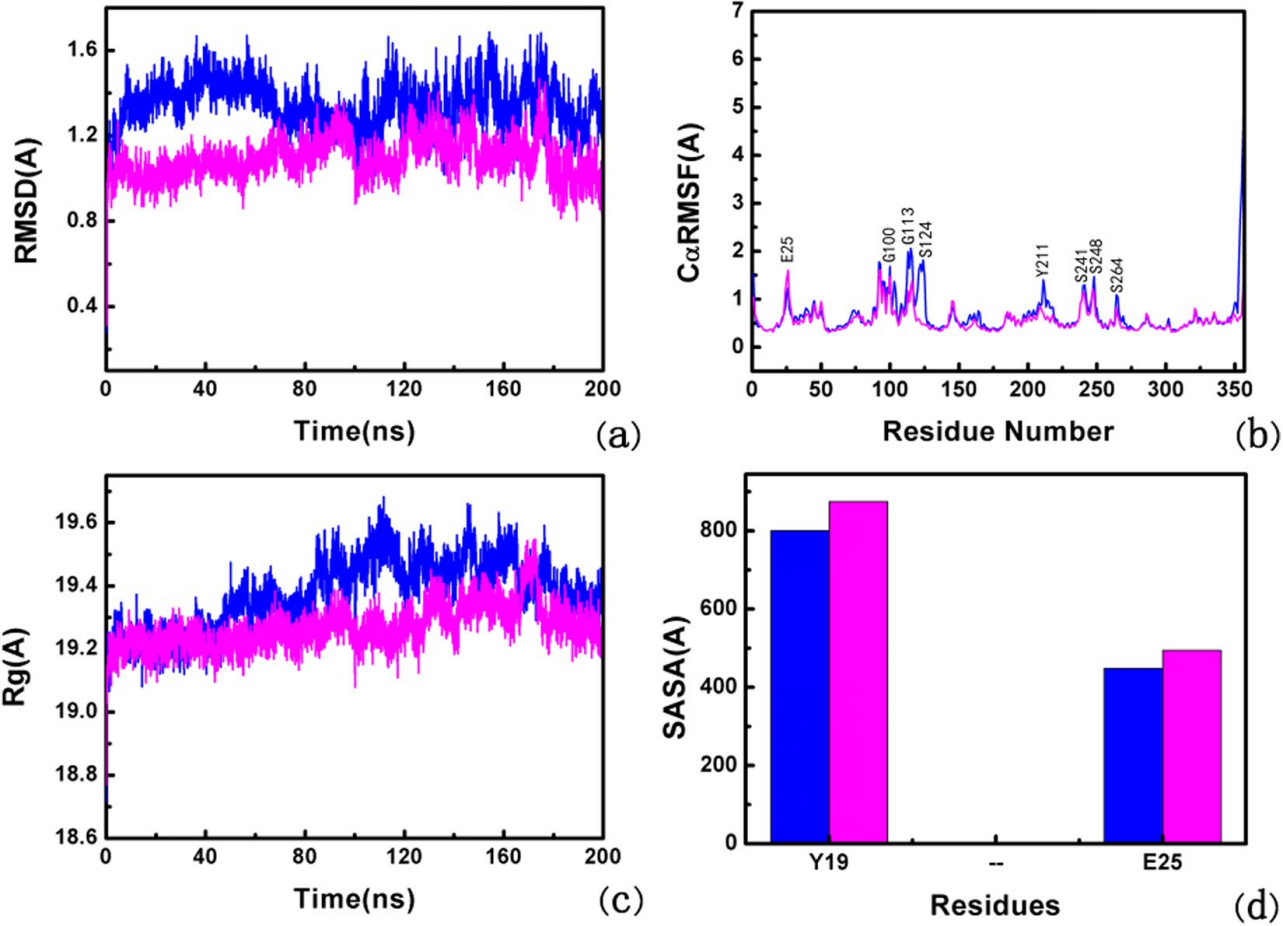
Conformational changes ADP-RA in the non-phosphorylated and phosphorylated ARH1. (**a**) RMSD plot of ADP-RA in the non-phosphorylated ARH1 (blue) and phosphorylated ARH1(purple) during 200 ns MD. (**b**) RMSF plot of ADP-RA in the non-phosphorylated ARH1 (blue) and phosphorylated ARH1(purple). (**c**) Radius of gyration (Rg) of ADP-RA in the non-phosphorylated ARH1 (blue) and phosphorylated ARH1(purple). (**d**) SASA for residues 19 and 25 of ADP-RA in the non-phosphorylated ARH1 (blue) and phosphorylated ARH1(purple).

In the study of ADP-RA with phosphorylated and non-phosphorylated ARH1 complexes conformations. The substrate (ADP-RA) was docked in ARH1 with AutoDock Vina. The compartment between the docked ligand conformation (blue) and the reference conformation in the crystal structure (red) located in the active site are shown in Fig. 1. The RMSD of the substrate (ADP-RA) is 1.923, which is similar to the RMSD of the reference ligand (=1.659). The root-mean-square (RMSD) analyses of protein backbone atoms and the ligand atomic coordinates were performed to measure the structure stability of the complexes. Figure 2(a), the RMSD variations of the two systems on 200 ns time scales’ MD simulation indicate that the complex atomic coordinates and the initial structures are similar, and the RMSD values of the two systems converge to 1.1 and 1.2 Å, respectively, which show the two systems are all stable.

The root-mean-square fluctuation (RMSF) of the backbone atoms of ADP-RA with phosphorylated and non-phosphorylated ARH1 complexes were analyzed to measure the mobility of the protein residues. Figure 2(b) shows that the large fluctuations of residues mainly occur in the ADP-RA with non-phosphorylated ARH1 complex connecting the regular secondary structure elements. Residues G100, G113, S124, Y211, S241, S248 and S264 have higher score in the ADP-RA non-phosphorylated ARH1 complex. The score is the Cα RMSF value. For the residue E25, the values which are 1.41 Å in the non-phosphorylated type and 1.42 Å in the phosphorylated type are similar. For the residues G100, G113, S124, Y211, S241, S248 and S264 in the non-phosphorylated type, the values which are 1.68 Å (G100), 1.99 Å (G113), 1.82 Å (S124), 1.41 Å (Y211), 1.30 Å (S241), 1.47 Å (S248), 1.09 Å (S264) are higher than the values in the phosphorylated type, which are 1.47 Å (G100), 1.17 Å (G113), 0.49 Å (S124), 0.72 Å (Y211), 1.08 Å (S241), 1.10 Å (S248), 0.82 Å (S264) respectively, which exhibit a high mobility during MD simulations. Figure 2(c) shows the radius of gyration (Rg) for the non-phosphorylated ARH1 (blue) and phosphorylated ARH1 (purple).

The mean Rg (The mean radius of gyration of residues during MD simulations was computed to test the flexibility of conformation quantitatively of protein) for the non-phosphorylated ARH1 is about 1.93 Å, while for the phosphorylated ARH1 is about 1.92 Å. The result in Fig. 2(c) shows that in the phosphorylated type, the ARH1 complex is less flexibility than in the non-phosphorylated type.

Time-dependent solvent-accessible surface area (SASA) has also been calculated for the ensemble of structures from the simulations Fig. 2(d). The solvent-accessible surface area of residues to find the points on a sphere that are exposed to solvent. we carried out the analysis of tunnels in protein (ARH1) by CAVER software. The results were shown in Figure S6 and Tables S1 and S2, indicate that Y19 and E25 are the residues in the tunnel-lining. Moreover Y19 is the phosphorylated site in the protein (ARH1) and E25 is able to form the intermediate α helix on the time scales simulation. So we selected these two residues to perform SASA calculation. The SASA of the hydrophilic residues (Y19 and E25) were performed as shown in Fig. 2(d). The area contributions for the non-phosphorylated type and the phosphorylated type show that the difference of the two residues folding degrees. For the phosphorylated type, the area contributions of Y19 and E25 are larger than in the non-phosphorylated complex, show the more active of two residues in the phosphorylated state. After a period of 200 ns, among the active pocket residues, the four phosphorylated residues and eight residues which have a higher RMSF score in MD simulations, Y19 and E25 have some changes in the phosphorylated ARH1.

**Figure 3:**
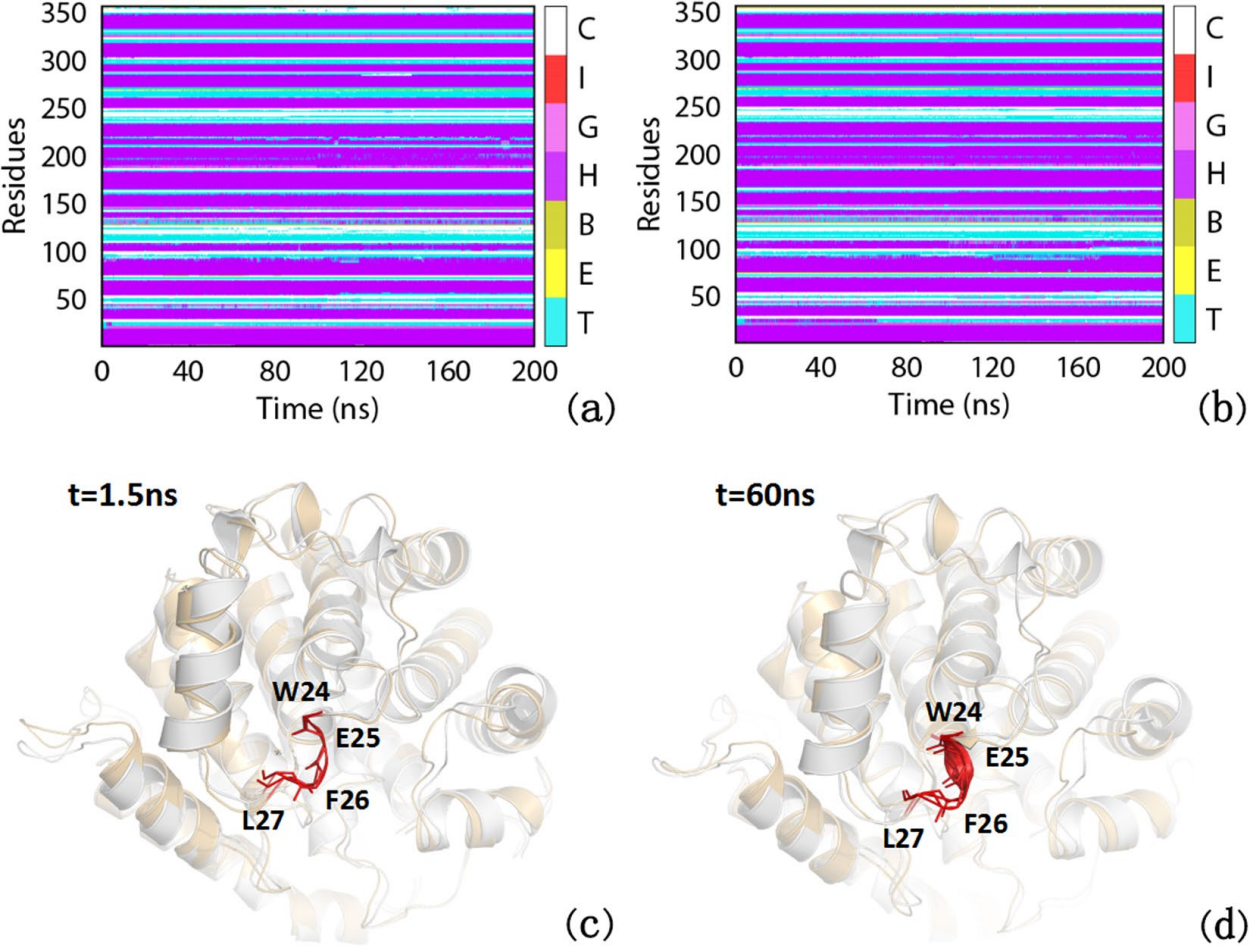
Dynamic changes of the secondary structure profile of ADP-RA in the ARH1 complex during 0~200 ns MD for (**a**) the non-phosphorylated type and (**b**) the phosphorylated type. The color bar represents different secondary structures as follow: 310 - helix (G), α - helix (H), π - helix (I), β - Bridge (B), β - bugle (E), turn (T), coil (C). (**c**) Structure alignment between non-phosphorylated complex (gray) and phosphorylated complex (yellow) after 1.5 ns MD; (**d**) Structure alignment between non-phosphorylated complex (gray) and phosphorylated complex (green) after 60 ns MD.

Subsequently, the secondary structure contents were also analyzed and the corresponding data was shown in Fig. 3(a–d), Figure S7 and Table 1. It can be seen that the α-helix content of non-phosphorylated ARH1 in E25 is 1.06% while in the phosphorylated ARH1 is about 25.88% (Table 1). The results of secondary structure analysis indicate that the short intermediate α helix of phosphorylated ARH1 during 200 ns MD simulations represents the ordered structure which may help the binding of ADP-RA to ARH1. Seen from Table S3 and Table S4, a hydrogen bond (Y19 and E25) disappear in the phosphorylated state, which may be useful to ADP binding.

**Table 1:** The α helix occupies during MD simulations.

Protein	Residue Number	Probability (α-helix)
non-phosphorylated ARH1	Glu 25	1.06%
phosphorylated ARH1	Glu 25	25.88%

**Figure 4:**
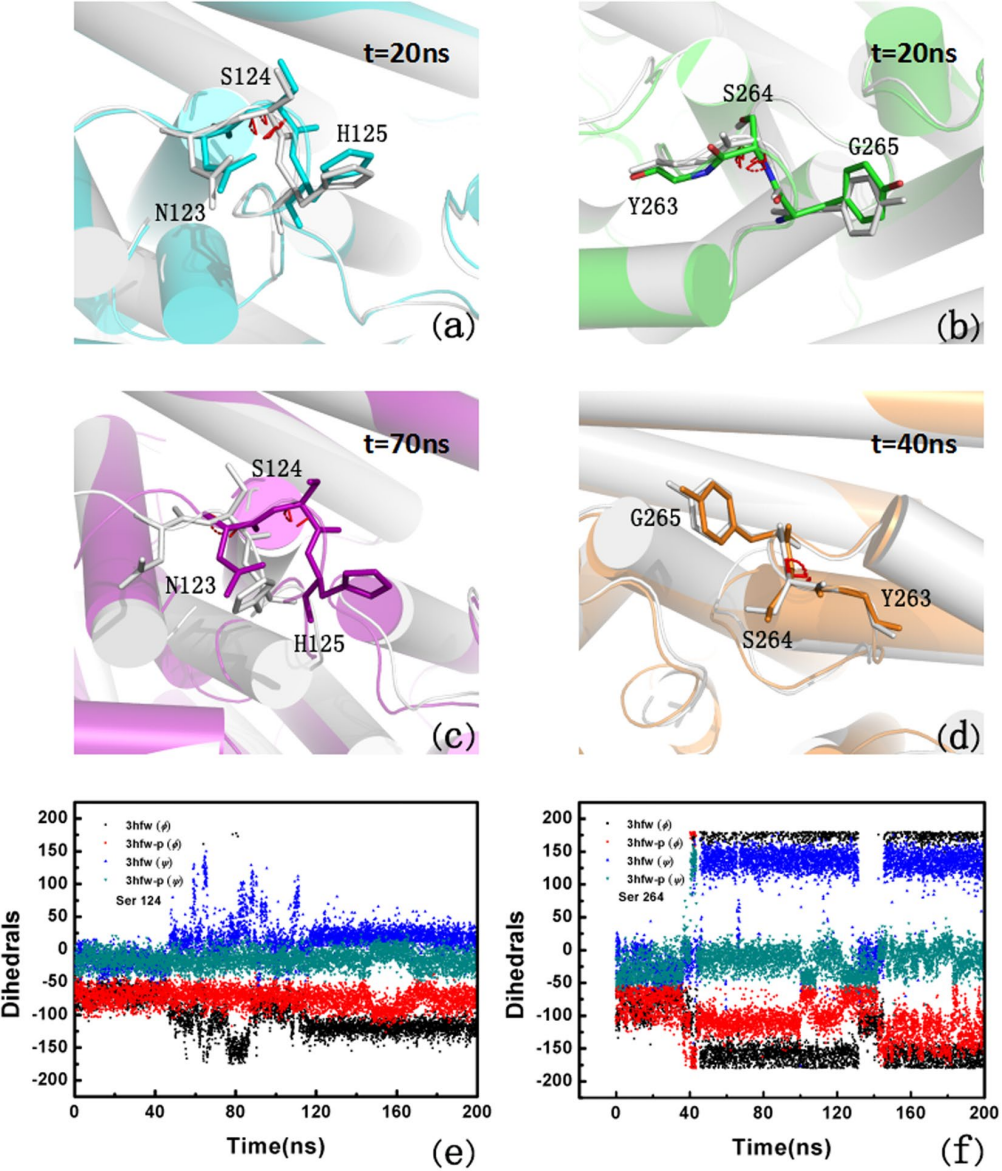
The dihedrals of S124 and S264 (**a**) S124 after 20 ns MD (gray represents non-phosphorylated-ADP-RA, same for b-d, and cyan represents for phosphorylated-ADP-RA); (**b**) S264 after 20 ns MD; (**c**) S124 after 70 ns MD; (**d**) S264 after 40 ns MD; (**e**) The dihedrals change of S124 of non-phosphorylated-ADP-RA and phosphorylated-ADP-RA complex during 200 ns MD; (**f**) The dihedrals change of S264 of non-phosphorylated-ADP-RA and phosphorylated-ADP-RA complex during 200 ns MD.

Seen from Figure S8, S124, D302, G100 and S264 are key residues in ADP-ribose binding pocket, which can make hydrogen bonds with ADP-ribose. The hydrogen bond occupancy between ADP-RA and residues of ARH1 were listed in Table 2. From Table 2, it can be seen that, the hydrogen bond occupancy between ADP-RA and D302 and G100 had not a large change, while the hydrogen bond occupancy between ADP-RA and S124 and S264 increased in the phosphorylated type. To qualify the rotation of S124 and S264, the variations of the mainchain of S124 and S264 (see Figure S9) were measured during the MD simulation, as shown in Fig. 4(a–f). The torsion angles of two residues all have a significant change non-phosphorylated ARH1, which indicate the two residues are disordered in the non-phosphorylated ARH1 type. The swing of ADP-RA may force S124 and S264 to undergo a displacement and thence to increase the hydrogen bond occupancy between ADP-RA and ARH in the phosphorylated type (Table 2), which can stabilize the complex.

**Table 2:** Hydrogen bond occupancies between ADP-RA and ARH1 during MD simulations.

Protein	ADP-RA	Occupancy (non-phosphorylated ARH1)	Occupancy (phosphorylated ARH1)
**Ser264:OH**	**O8**	17.08%	61.99%
**Ser124:O**	**H**	15.32%	48.41%
**Gly100:NH**	**O12**	0.14%	0.4%
**Asp302:OH**	**O12**	0.02%	0.02%

Reliable free energy calculations based on molecular dynamics (MD) simulations can provide the missing links between experimental binding affinities and 3D structures of protein-ligand complexes. When ligands differ by larger substitutes, or receptors differ by more drastic mutations (e.g., to alanine, alanine scanning calculation), it can be used to test the performance of the residues. Alanine scanning^38^ is a direct way of mutagenesis with A replacing the special residues. In this study, alanine scanning calculation was used to S124 and S264, and the results were listed in Table 3 and Figure S10. The results show that in the non-phosphorylated/ phosphorylated type, the binding energies of the residues (S124 and S264) are lower than the energies of the mutations (S124A and S264A), thus S124 and S264 have more close interactions with the ADP-RA than mutations (to alanine). Moreover, comparing the binding energy of the phosphorylated type with ADP-RA complex to the non-phosphorylated type, the calculation shows that the binding energies of the residues (S124 and S264) in the phosphorylated are lower than in the non-phosphorylated, which can help the binding to the ADP-RA.

**Table 3:** The MM-GBSA results of the four residues (Ser124, Ser264, S124A and S264A).

	ΔE_ele_	ΔE_vdw_	ΔG_sol_np_	ΔG_sol_polar_	ΔG_polar_	ΔG_nonpolar_	ΔG_bind_
SER124-ADP-RA(NPS^a^)	−0.090	−0.029	0.000	0.121	0.031	−0.029	0.002
SER264-ADP-RA(NPS)	−0.745	−0.166	−0.021	0.876	0.131	−0.187	−0.056
SER124-ADP-RA(PS^b^)	−2.706	−1.016	−0.241	3.399	0.693	−1.257	−0.563
SER264-ADP-RA(PS)	−0.385	−0.351	−0.046	0.433	0.048	−0.397	−0.349
ALA124(NPS^a^)	−0.233	−0.050	−0.000	0.299	0.066	−0.050	0.016
ALA264(NPS)	−0.690	−0.173	−0.018	0.981	0.291	−0.191	0.104
ALA124(PS)	−0.502	−0.400	−0.041	0.450	−0.052	−0.441	−0.493
ALA264(PS)	−0.769	−0.383	−0.021	0.842	0.073	−0.404	−0.331

^a^NPS: Non-phosphorylated state. ^b^PS: Phosphorylated state.

**Figure 5:**
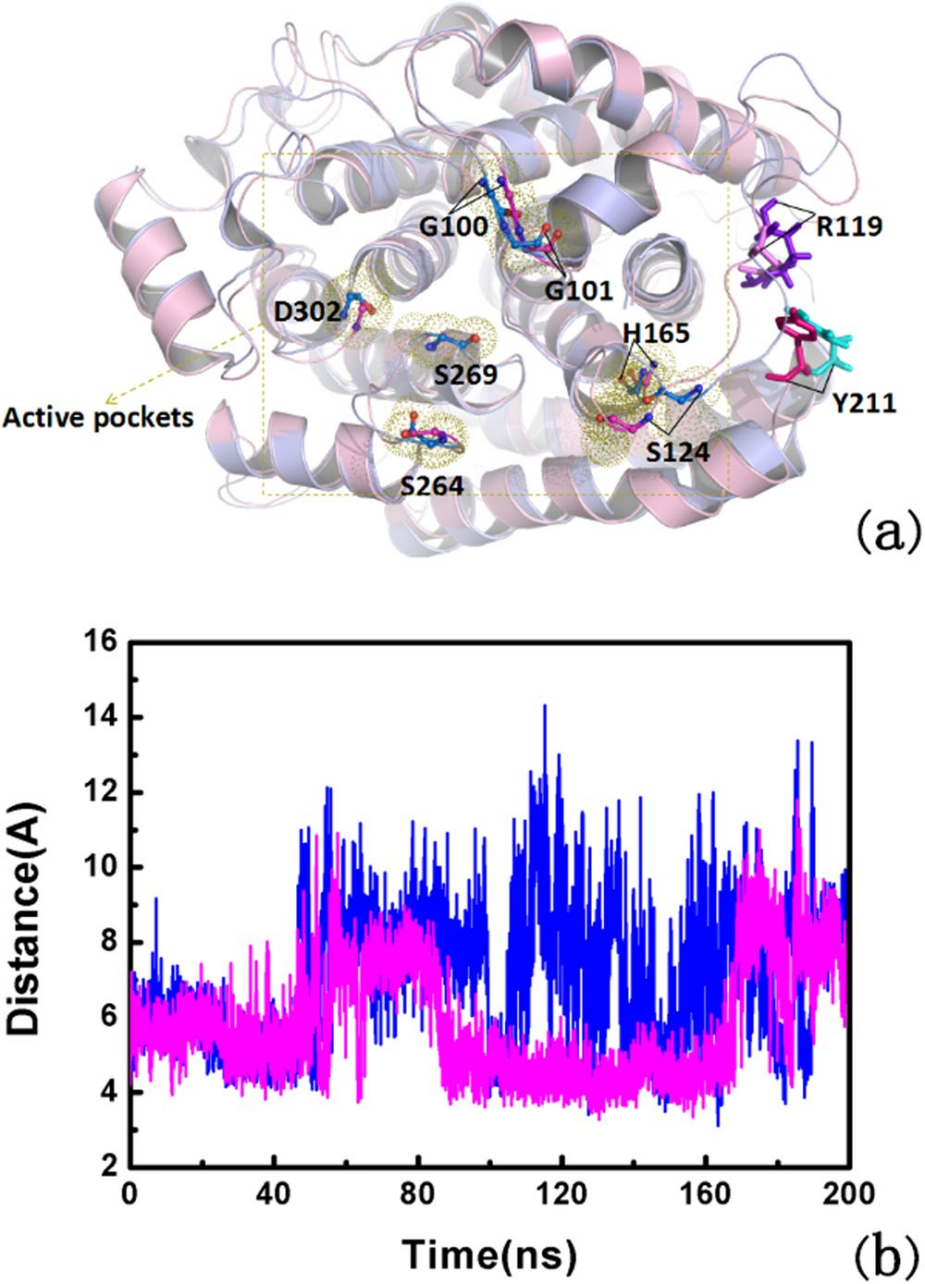
Distance between the centroid of Y211 and the guanidino group of R119: (**a**) The conformation of Y211 and R119, the pink structure is phosphorylated, and the residues of the binding pocket; (**b**) Dynamics during 200 ns MD for the non-phosphorylated ARH1-ADP-RA (blue) and phosphorylated ARH1-ADP-RA (purple).

According to the previous reports, the cation–π interaction is regarded as a key interaction in the protein structure^39^. As demonstrated in Table 4, the cation–π interactions in ARH1 were calculated. R119 and Y211 show the strongest cation–π interactions in ARH1 as calculated by using the Realistic Electrostatics program^39,40^. Figure 5(a) and (b) indicate that most of the distances between R119 and Y211 (from the cation to the centroid of the aromatic ring) are more than 5 Å in the phosphorylated ARH1-ADP-RA, showing that the interaction between R119 and Y211 is more stable in the phosphorylated ARH1 system. For demonstrated the relation between the less flexibility of the residue in the simulation with the binding of ADP-RA. We supplied the distances between R119 and Y211 (from the cation to the centroid of the aromatic ring) on 200 ns time scales. As shown in Fig. 5, the residues R119 and Y211 are located near to the loop of the active substrate binding pocket. These structural changes near to the loop of the binding pockets in the phosphorylated ARH1 may useful to substrate binding. In additional, from Tables S3 and S4, hydrogen bonds among Y205 become more in the phosphorylated state than that of non- phosphorylated state. Hydrogen bonds may be important for protein stability, which are useful to ADP binding.

**Table 4:** Cation-π interaction in ARH1.

Cation-π	*E*_(es)_ (kcal/mol)	*E*_(vdw)_ (kcal/mol)
R141-F142	−2.07	−2.67
R234-F245	−1.18	−2.65
R234-F257	−2.09	−1.29
R254-F249	−2.19	−1.05
R119-Y211	−3.53	−3.32
R134-Y87	−2.97	−3.02
R186-Y181	−3.89	−2.58
R336-Y333	−3.45	−1.95
R119-W118	−2.91	−1.68
K203-W220	−2.6	−1.29

To sum up, the phosphorylations on ARH1 lead three structural changes as follow: (1) E25 is more frequently in the α-helix conformationin the phosphorylated type with ADP-RA complex (51.56%) than that of the non-phosphorylated complex (2.12%); (2) S124 and S264 become less flexible in the phosphorylated type with the ADP-RA complex, which is helpful for the two residues formhydrogen bonds with ADP-RA; (3) Y211 is also less flexible in the phosphorylated type with ADP-RA complex, and it is useful for the stability of cation-π interaction of Y211-R119. The significance of difference between the phosphorylated and non-phosphorylated proteins lay in the phosphorylation of four residues can disrupt or form some H-bonds and lead to conformational changes in the active pocket, which may help the ligand binding and Mg^2+^ coordination, and thus can affect the catalytic efficiency of ARH1.

### Principal Component Analysis

**Figure 6:**
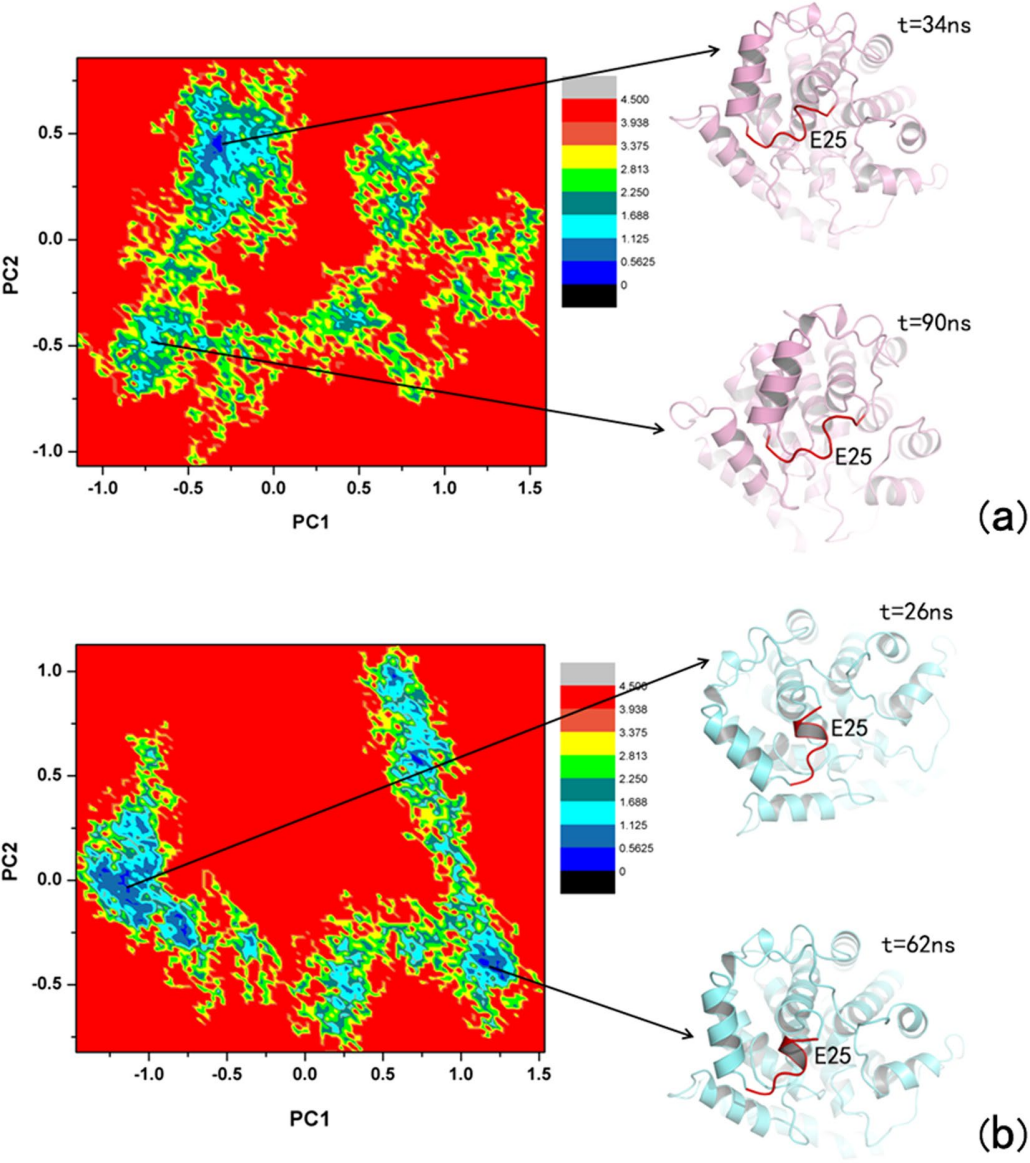
Examples of free energy landscape for (**a**) non-phosphorylated-ADP-RA complex, and (**b**) phosphorylated-ADP-RA complex.

The Free Energy Landscape (FEL), whose representation is achieved by projecting the trajectories on their first two principal components of motion^41–44^ are shown in Fig. 6(a) and (b). The first two principal components of motion are the considered reaction-coordinates. From this analysis, the minimum frequency value^45^, or the probability of the most likely conformation, and the number of minima over a case dependent threshold of frequency^46^.

The conformational transitions of the E25 are reflected in different regions of the free energy landscape (FEL), which is depicted on the basis of the projection of the first two principal components of the Cα trajectory (Fig. 6(a) and (b)). As shown in Fig. 6(a), the lowest-energy region of the FEL ofthe phosphorylated ARH1-ADP-RA is achieved after 26 ns, while the non-phosphorylated ARH1-ADP-RA complex does not become stable until 34 ns (Fig. 6(b)). In addition, the low-energy zone of the phosphorylated ARH1-ADP-RA is larger than that of the non-phosphorylated ARH1-ADP-RA, which suggests that the non-phosphorylated ARH1 protein undergoes a relatively long transition stage to reach the equilibrium. The dynamic conformational changes are driven by modifications (in the phosphorylated ARH1-ADP-RA complex, E25 located at α helix, while in the non-phosphorylated ARH1-ADP-RA complex, E25 located in the loop), mainly phosphorylation, to the four tyrosine residues.

The first few eigenvalues correspond to the concerted motions, and their amplitudes decrease to reach a large number of constrained but more localized fluctuations^47^. The first two principal components account for 37.36 and 42.11% of the motion observed in 200 ns of the trajectories for non-phosphorylated ARH1-ADP-RA, and phosphorylated ARH1-ADP-RA, respectively (Table 5), which reveals that the properties of the motions described by the first few principal components are different to non-phosphorylated ARH1-ADP-RA, and phosphorylated ARH1-ADP-RA as the magnitudes of the eigenvalues are higher for phosphorylated ARH1-ADP-RA, suggesting that phosphorylated ARH1 is the most affected by the presence of ADP-RA.

**Table 5:** Principle component probability during MD simulations.

Protein	Principle component (PC)	Probability
Non-phosphorylated ARH1	PC1	37.36%
	PC2	20.73%
Phosphorylated ARH1	PC1	42.11%
	PC2	15.82%

### SMD study

**Figure 7:**
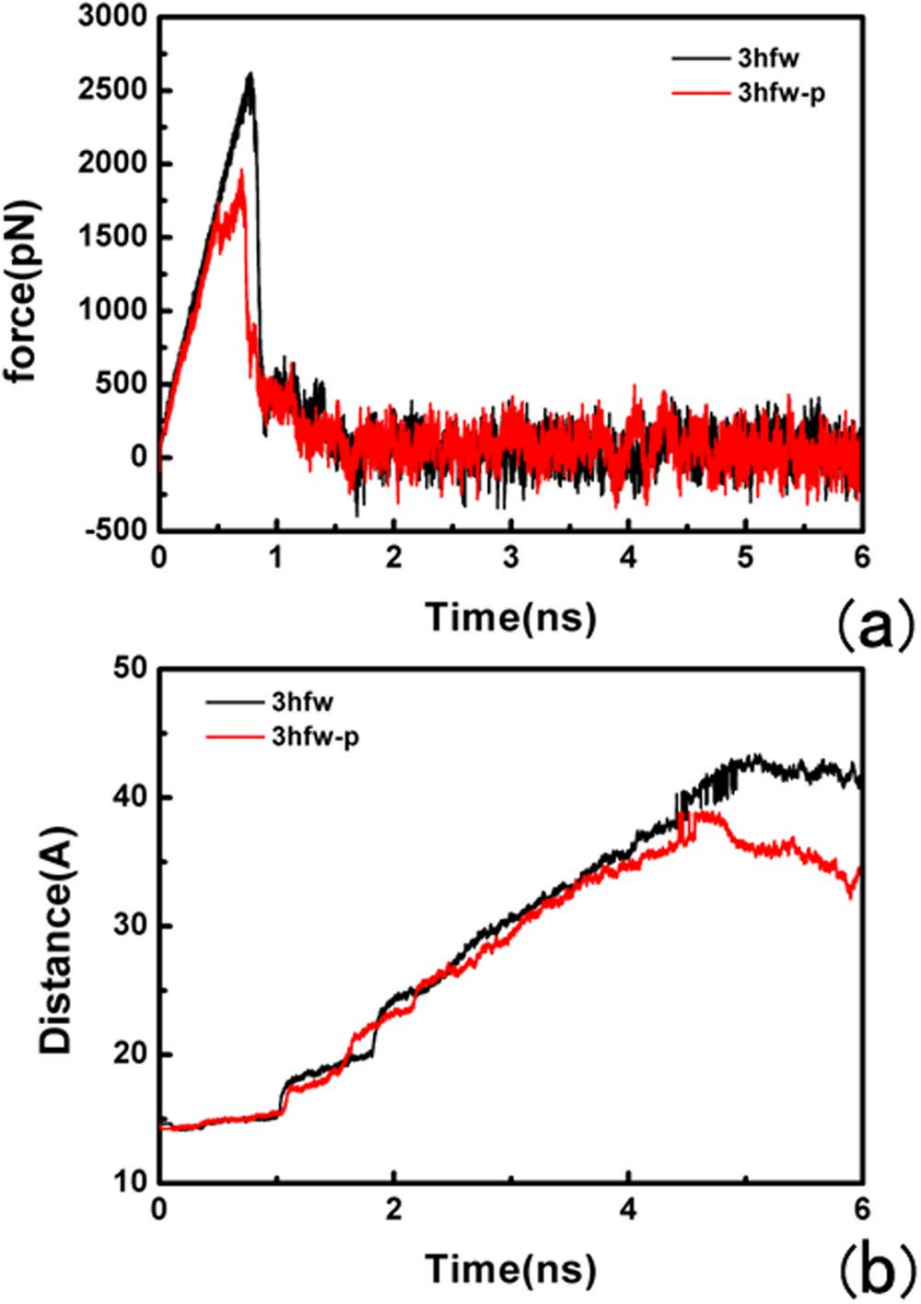
SMD simulation results. (**a**) Typical force profiles of ADP-ribose pulled out of the binding pocket along the unbinding pathway (non-phosphorylated ARH1 (black), phosphorylated ARH1 (red)). (**b**) Time dependence of the distance change for (non-phosphorylated ARH1 (black), phosphorylated ARH1 (red) in the two SMD simulations.

SMD^45^ can provide qualitative insights into the interactions and conformational changes between the ligand and its surrounding residues when inducing unbinding of ligand along the pathway on the molecular dynamics simulation. The samples for the same unbinding pathway are more extensive. In this study of SMD simulation, we performed the representative force profiles of the ligand, as showed here, Fig. 7(a) and (b) indicate the force profiles for the ADP-ribose specific unbinding from the binding pocket of phosphorylated ARH1 and non-phosphorylated ARH1. As demonstrated here, in Fig. 7(a), the time-dependent external pulling force applied to ADP-ribose with non-phosphorylated ARH1 increased linearly in the initial stage of the SMD simulation. Afterward, at around 700 ps, the pulling force reached a peak value of just about 2621 pN. At this moment, ADP-ribose was about to leave the active site of the non-phosphorylated ARH1. The value of the external force then rapidly decreased to zero and featured minor fluctuations near zero. Figure 7(a) indicates that the pulling force exerted on ADP-ribose with phosphorylated ARH1 increased directly in the first stage on the time scale SMD simulation until around 736 ps, at this time the pulling force reached a value of 1965 pN. The differences of the pulling force reflect the conformational changes ADP-ribose in the solvent, describing its full dissociation from the binding pocket of the protein.

**Figure 8:**
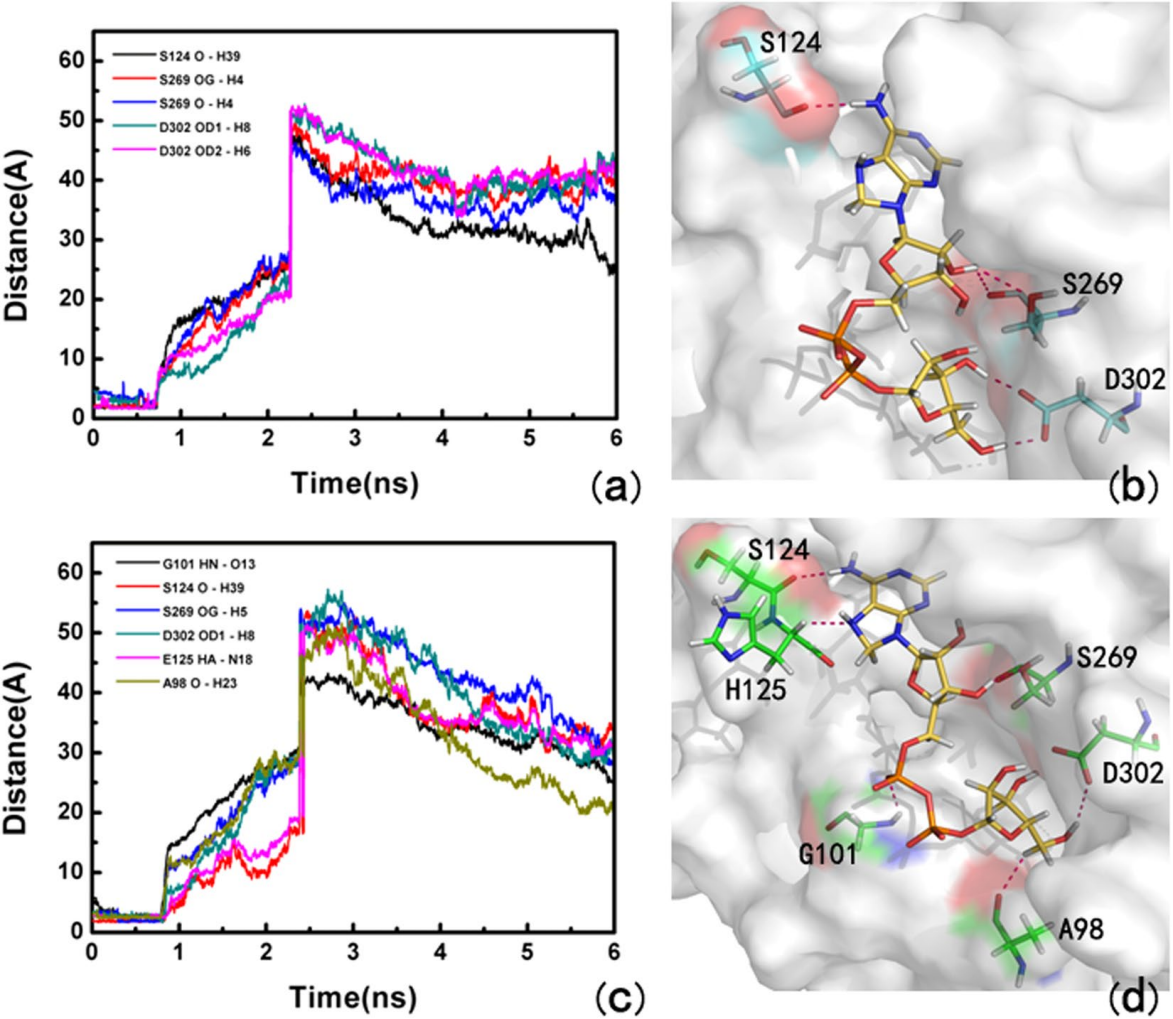
Hydrogen bond distance between ADP-ribose and the binding pocket of ARH1 calculated from the centroid of the aromatic ring pulled out of ARH1, (**a**,**b**) for the non-phosphorylated ARH1 and (**c**,**d**) the phosphorylated ARH1.

**Figure 9:**
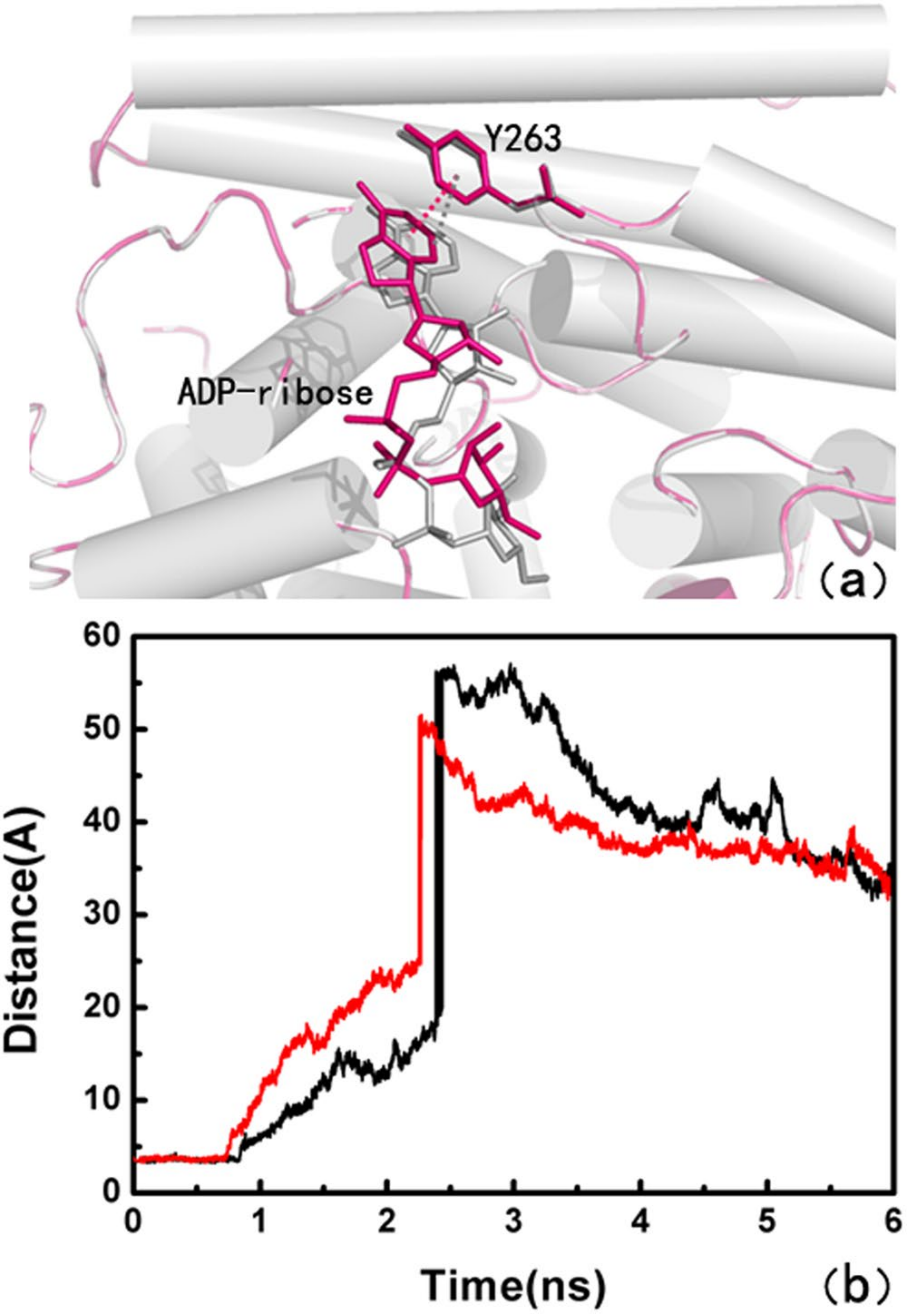
Distance of the cation-π interaction with Y263: non-phosphorylated ARH1 (**a**, black), phosphorylated ARH1 (**b**, red) in the two SMD simulations.

At the beginning for the X-ray structure and thence used for SMD, ADP-ribose had literally tight interactions with its surrounding residues including S124, H165, S264, G101, G100, G127, G128, E25, D302, S270 and S269 by the strong hydrogen bonding and the cation-π interaction with Y263 at t = 0 ps with non-phosphorylated ARH1-ADP-ribose complex (see Figure S11). After ~700 ps for the non-phosphorylated ARH1-ADP-ribose complex, some hydrogen bonds were broken thus the interactions of the ligand with non-phosphorylated ARH were weakened. At this stage, S269, S124, and D302 formed hydrogen bonds with ADP-ribose (Fig. 8(a) and (b). The cation-π interaction between ADP-ribose and Tyr263 during SMD are shown in Fig. 9(a) and (b). Interestingly, although the ligand had a ~4.0 Å fluctuation align to its initial position, the strong hydrogen bonding with S264 with ADP-ribose still existed (t = 900 ps; see Figure S11) and disappeared at ~960 ps.

At the beginning for the X-ray structure and thence used for SMD, ADP-ribose had literally tight interactions with its surrounding residues including S124, H125, G127, G128, G101, G100, D55, A98, B302, S269 and S264, and the cation-π interaction with Y263 at t = 0 ps with phosphorylated ARH1-ADP-ribose complex (see Figure S11). After ~736 ps for the non-phosphorylated ARH1-ADP-ribose complex, S124, H125, G101, A98, D302, and S269 formed hydrogen bonds with ADP-ribose (Fig. 8(c) and (d). Compared with ADP-ribose with phosphorylated ARH1 and non-phosphorylated ARH1 type, it can be seen that E25 does not appeared at the unbinding pathway in the phosphorylated ARH1 and cannot hinder ADP-ribose leaving.

The average values of F_max_ and F_sum_ based on ten SMD trajectories are presented in Table 6. The F_sum_ value is calculated based on the saved data points, for avoiding deviation of its real energy, an arbitrary unit is performed in the average values calculation^44^. An arbitrary unit is given to avoid deviation of its real energy. In this study of the average values, either F_max_ or F_sum_ supports phosphorylated ARH1, which is favorable for ADP-ribose to be detached from ARH1.

**Table 6:** Average Values of F_max_ and F_sum_.

Pathways	F_max_ (pN)	F_sum_ (pN)
Path 1	2621 ± 51	1.48 × 10^7^ ± 777
Path 2	1965 ± 37	1.13 × 10^7^ ± 556

### PMF and MM-GBSA calculations

**Figure 10:**
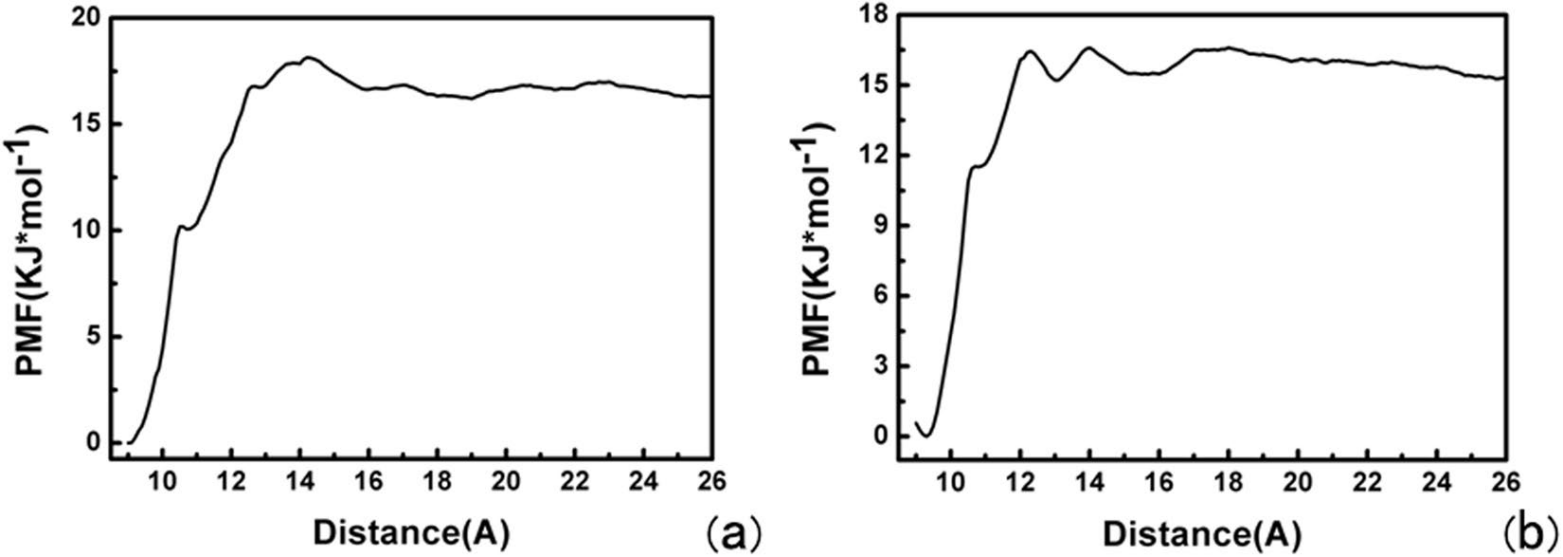
PMF profile along the unbinding pathway of (**a**) the non-phosphorylated ARH1 and (**b**) the phosphorylated ARH1.

In the following we performed the reconstruction of a potential of mean force (PMF) from SMD data.Based on the SMD trajectories, we separated a reaction coordinate into 12 equal sections. As shown in Fig. 10(a) and (b), the free energy differences for the two approaches of ligand-receptor complexes in PMF method were calculated. From the PMF calculated profiles, something interesting about the dissociation from the binding pocket of the ARH1 were revealed. At the initial stage, when the ligand depart from the initial conformational position, the free energy curve displayed a linearly increase, and this free energy demonstrated the interaction between the ligand and the binding pocket residues. The differences of free energy curve between non-phosphorylated with phosphorylated complexes are indicated in Fig. 10, for ADP with non-phosphorylated ARH1 in Fig. 10(a), the initial conformation of the inhibitor was in the considerable stable binding mode with the ARH1. Conversely, in Fig. 10(b), with the departure of the inhibitor from the protein binding pocket, the free energy value rapidly increased, which can be due to the interaction of the inhibitor with the active site residues. The free energy central barrier of the inhibitor unbinding process for the phosphorylated ARH1 is around 16.61 kcal·mol^−1^ while for the non-phosphorylated ARH1 it is around 18.14 kcal· mol^−1^. Thus, non-phosphorylated ARH1 is easy for ADP-ribose to be taken off.

The binding free energy calculated from the MM-GBSA is shown in Table 7, the results list ligand affinity with enzyme. For testing the binding free energy of ARH1-ADP-ribose complex, we extracted a representative comformation and a peak-timing structure on SMD simulation time scales. As shown in Table 7, the binding energies of the non-phosphorylated ARH1-ADP-ribose are −11.05 kcal·mol^−1^ (representative ARH1-ADP-ribose) and −18.33 kcal·mol^−1^ (ARH1-ADP-ribose-Peak) while for the phosphorylated ARH1-ADP-ribose are −23.57 kcal·mol^−1^ (representative ARH1-ADP-ribose) and −24.00 kcal·mol^−1^ (ARH1-ADP-ribose-Peak). The phosphorylated ARH1-ADP-ribose has lower binding energies, thus the ADP-ribose binding to ARH1 is more stable.

**Table 7:** The MM-GBSA results.

	ΔE_ele_	ΔE_vdw_	ΔG_sol_np_	ΔG_sol_polar_	ΔG_polar_	ΔG_nonpolar_	ΔG_bind_
Non-phosphorylated ARH1-ADP-ribose	216.03	−36.20	−4.72	−193.43	22.59	−40.93	−11.05
phosphorylated ARH1-ADP-ribose	433.50	−36.36	−4.10	−404.09	29.41	−40.46	−18.33
Non-phosphorylated ARH1-ADP-ribose -Peak	213.50	−44.64	−5.24	−187.19	26.31	−49.88	−23.57
phosphorylated ARH1-ADP-ribose-Peak	440.69	−50.49	−5.02	−409.17	31.51	−55.51	−24.00

## Conclusion

ADP-ribosylation is a reversible and covalent PTM. Until now, the mechanism of PTM of ARH1 and phosphorylationinduced the ligands binding and unbinding processions is still poorly understood. MD simulations were used and the results indicate that E25 is more frequently in the α-helix conformationin the phosphorylated with ADP-RA complex than that of the non-phosphorylated complex, S124 and S264 become less flexible in the phosphorylated with ADP-RA complex, and Y211 is also less flexible in the phosphorylated with ADP-RA complex, in which these changes are useful for ADP-RA to bind the phosphorylated ARH1. The possible unbinding pathways of ADP-ribose from non-phosphorylated and phosphorylated ARH1 were explored by using SMD simulations. The results have showed phosphorylated ARH1 has more ordered structure than that of non-phosphorylated type, which can induce ligands to make more stable interactions ether in the SMD simulations. Our results will be useful for further studying PTM-induced ARHs structural change study.

## Electronic supplementary material

supplementary information

The trajectory of SMD simulation for the non-phosphorylated

The trajectory of SMD simulation for the phosphorylated
