# Supplementary material for: Understanding the differences of the ligand binding/unbinding pathways between phosphorylated and non-phosphorylated ARH1 using molecular dynamics simulations
Source: Sci Rep. 2017 Sep 29;7:12439. doi: 10.1038/s41598-017-12031-0 (PMC5622063; doi:10.1038/s41598-017-12031-0)
Supplement: Supplementary file 1 — supplementary information [file 41598_2017_12031_MOESM1_ESM.pdf]

Understandingthe differences of the ligand binding/unbinding pathways between phosphorylated and non-phosphorylated ARH1 using molecular dynamics simulations

Jingxuan Zhu[a], YishuoLv[a], Xiaosong Han[b,c], Dong Xu[b,c]*, and Weiwei Han[a,b*]

Figure S1 The reaction catalyzes by ARH1.

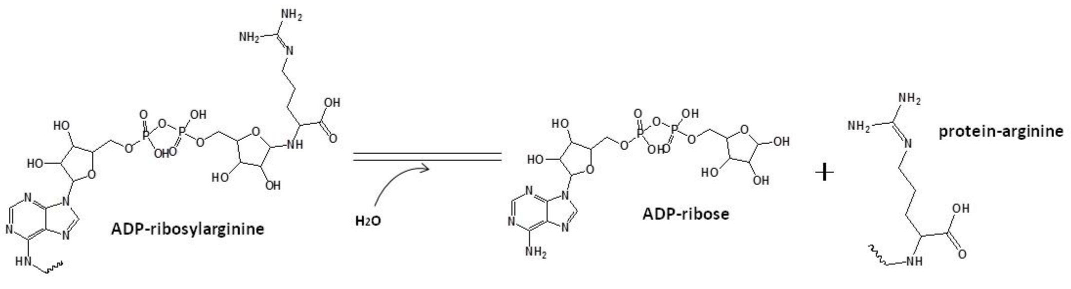

Figure S2 RMSD plot of ADP-RA in the non-phosphorylated ARH1 for three MD simulations.

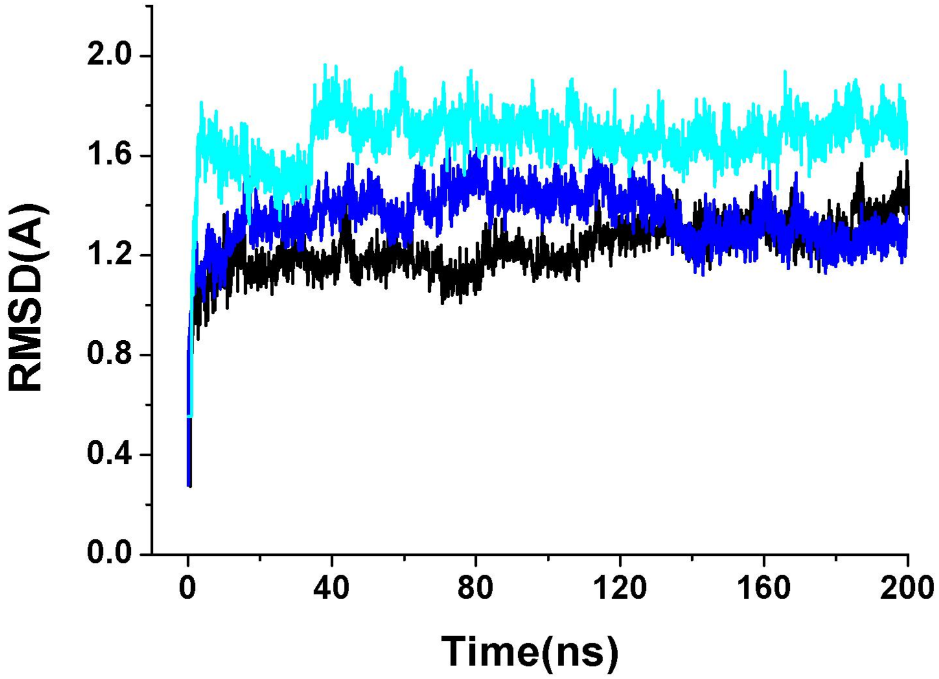

Figure S3 RMSD plot of ADP-RA in the phosphorylated ARH1 for three MD simulations.

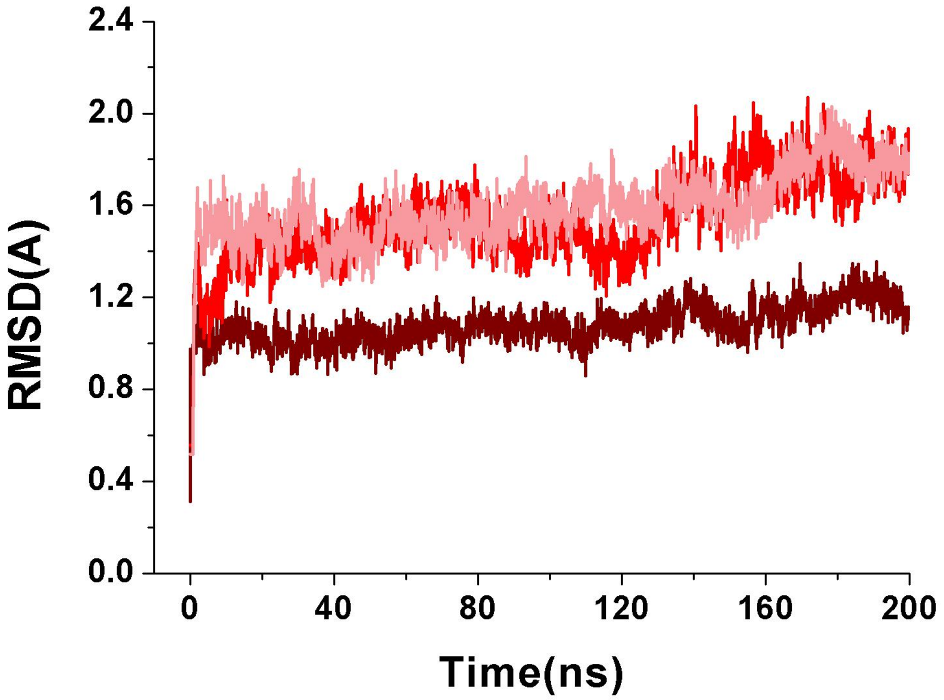

Figure S4 The force profiles of three SMD simulations along the unbinding pathways (non-phosphorylated ARH1).

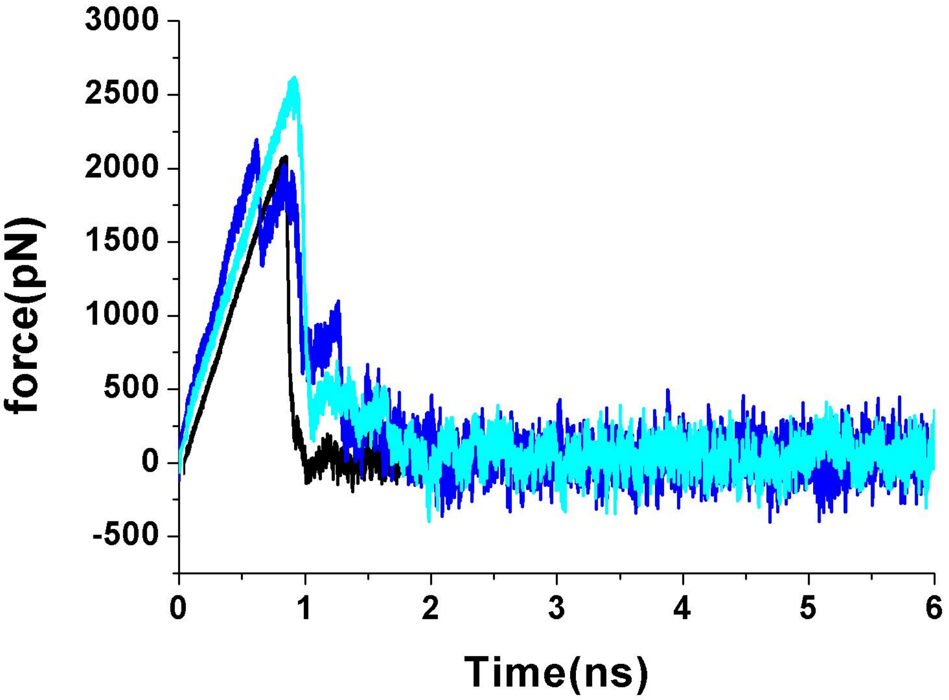

Figure S5 The force profiles of three SMD simulations along the unbinding pathways (phosphorylated ARH1).

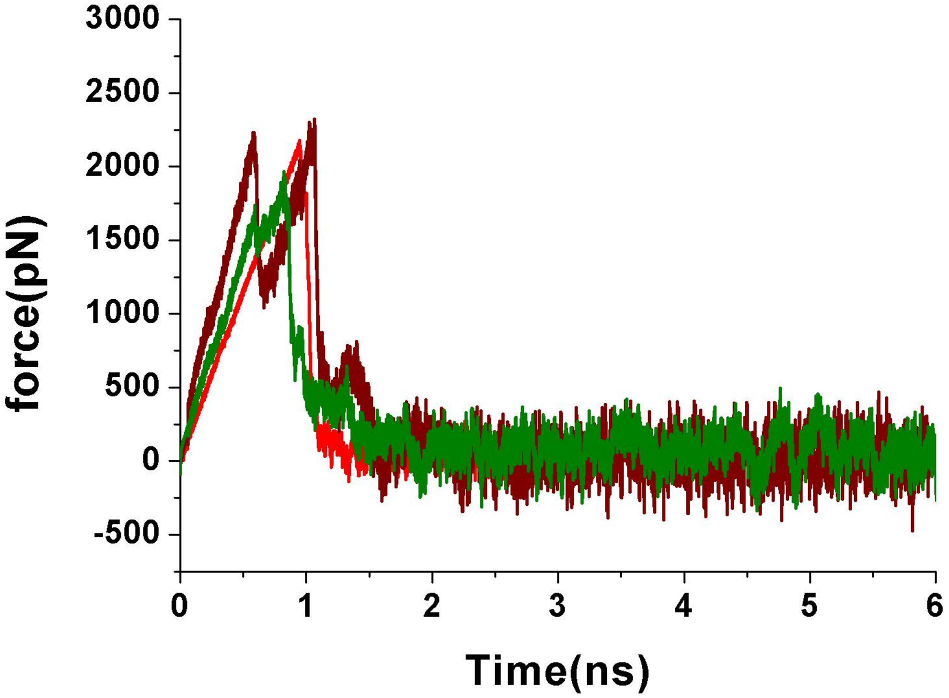

Figure S6 The typical tunnel-lining residues (a) for the non-phosphorylated, (b) for the phosphorylated.

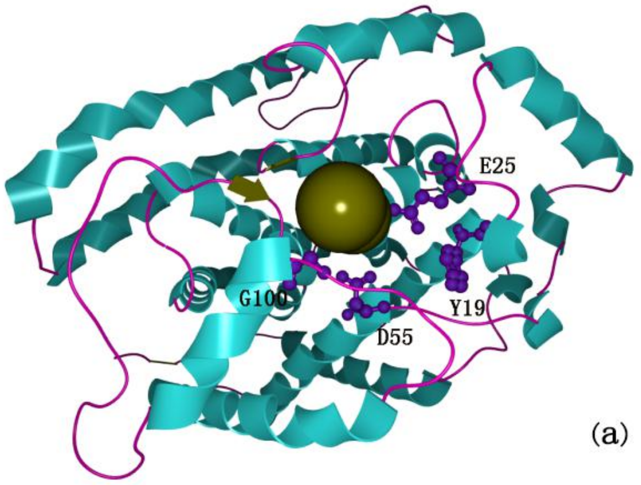

(a)

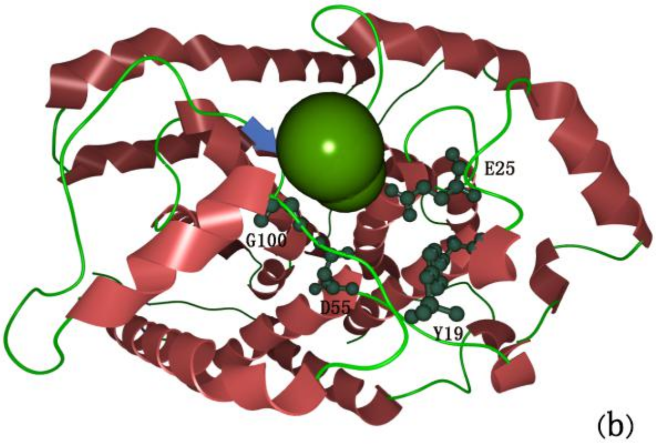

(b)

Figure S7 a Dynamic changes of the secondary structure profile of ADP-RA in the ARH1 complex during 100-200 ns MD: (a) for the non-phosphorylated typeand and (b) the phosphorylated type. The color bar represents different secondary structures as follow: 310 - helix (G), α - helix (H), π - helix (I), β - Bridge (B), β - bugle (E), turn (T), coil (C).

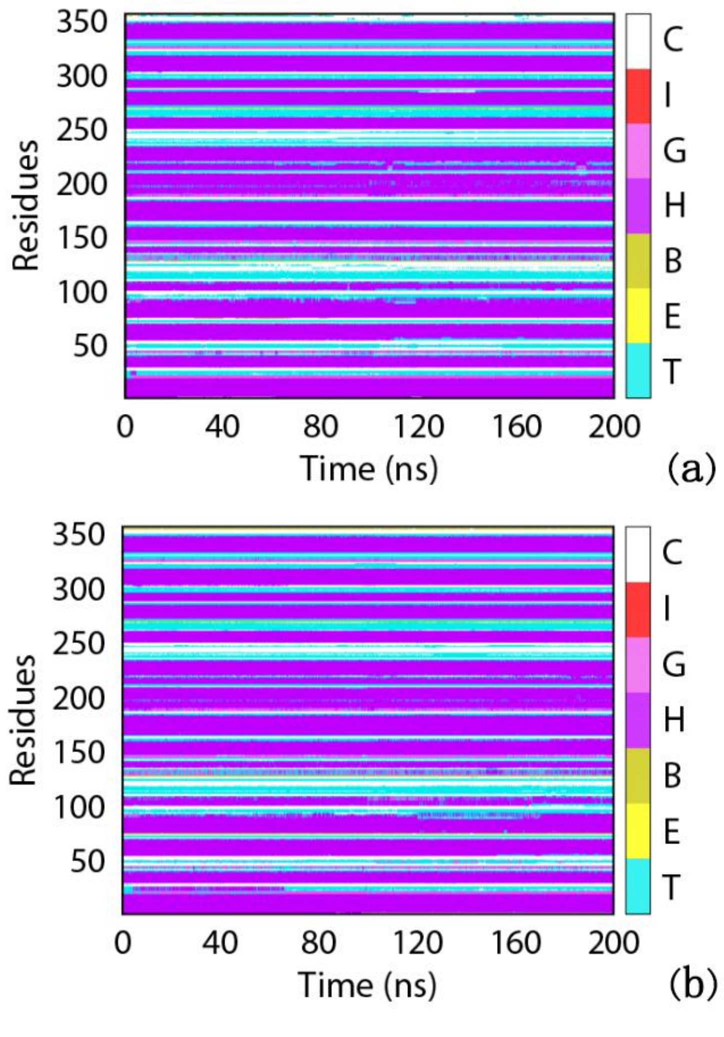

Figure S8 ADP-ribose in the ARH1 active pocket.

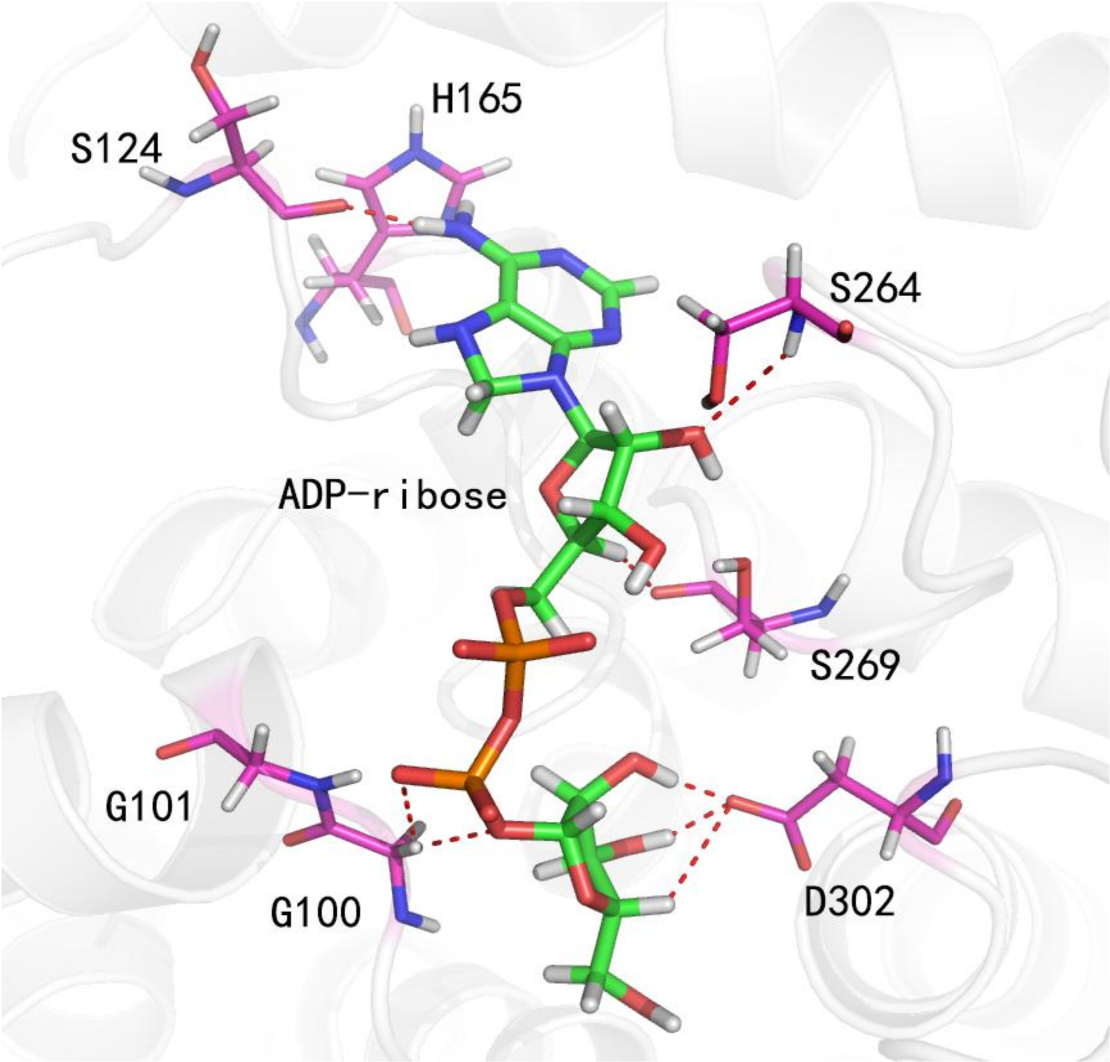

Figure S9 The chemical structure of the two residues (S124 and S264) and the four torsion angles.

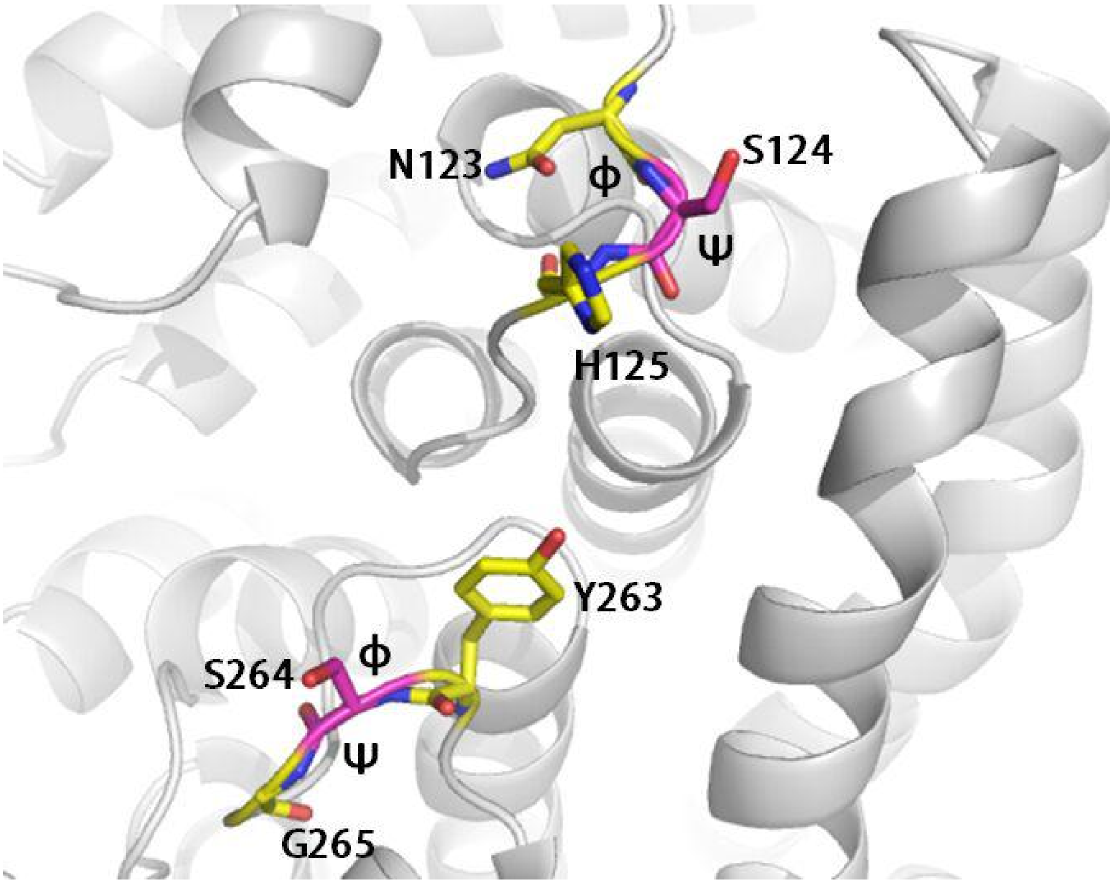

Figure S10 The MM-GBSA results of the four residues (Ser124, Ser264, S124A and S264A). NPS: Non-phosphorylated state, PS: Phosphorylated state.

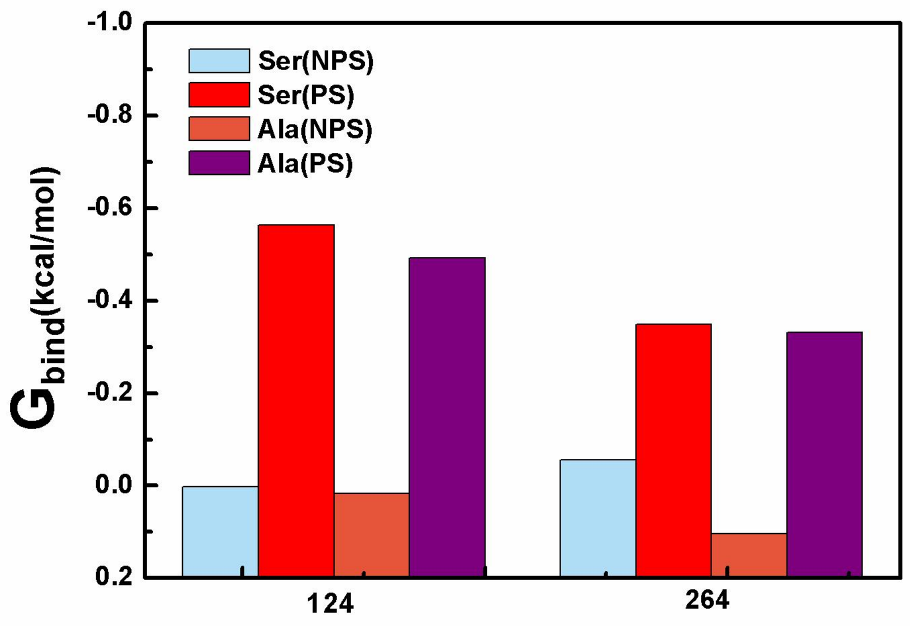

Figure S11 Conformational changes during SMD.

(a) non-phosphorylated-ADP-ribose complex (b) phosphorylated-ADP-ribose complex.

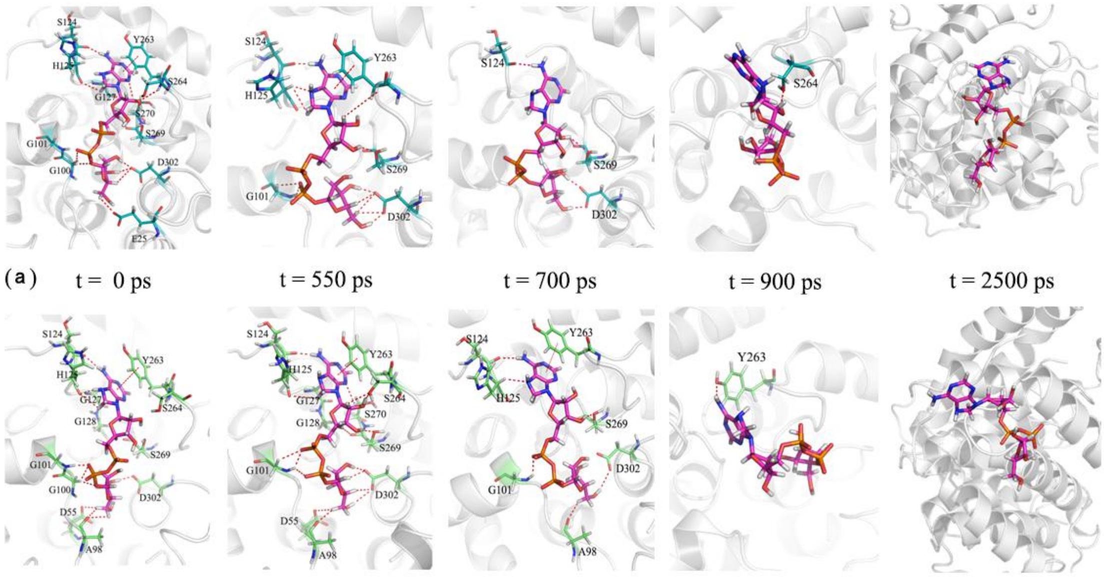

(a) t = 0 ps t = 550 ps t = 700 ps t = 900 ps t = 2500 ps

(b) t = 0 ps t = 550 ps t = 736 ps t = 1000 ps t = 2500 ps

Figure S12 The diagram of the pathway of SMD simulations.(a) for the non-phosphorylated type and (b) for the phosphorylated type.

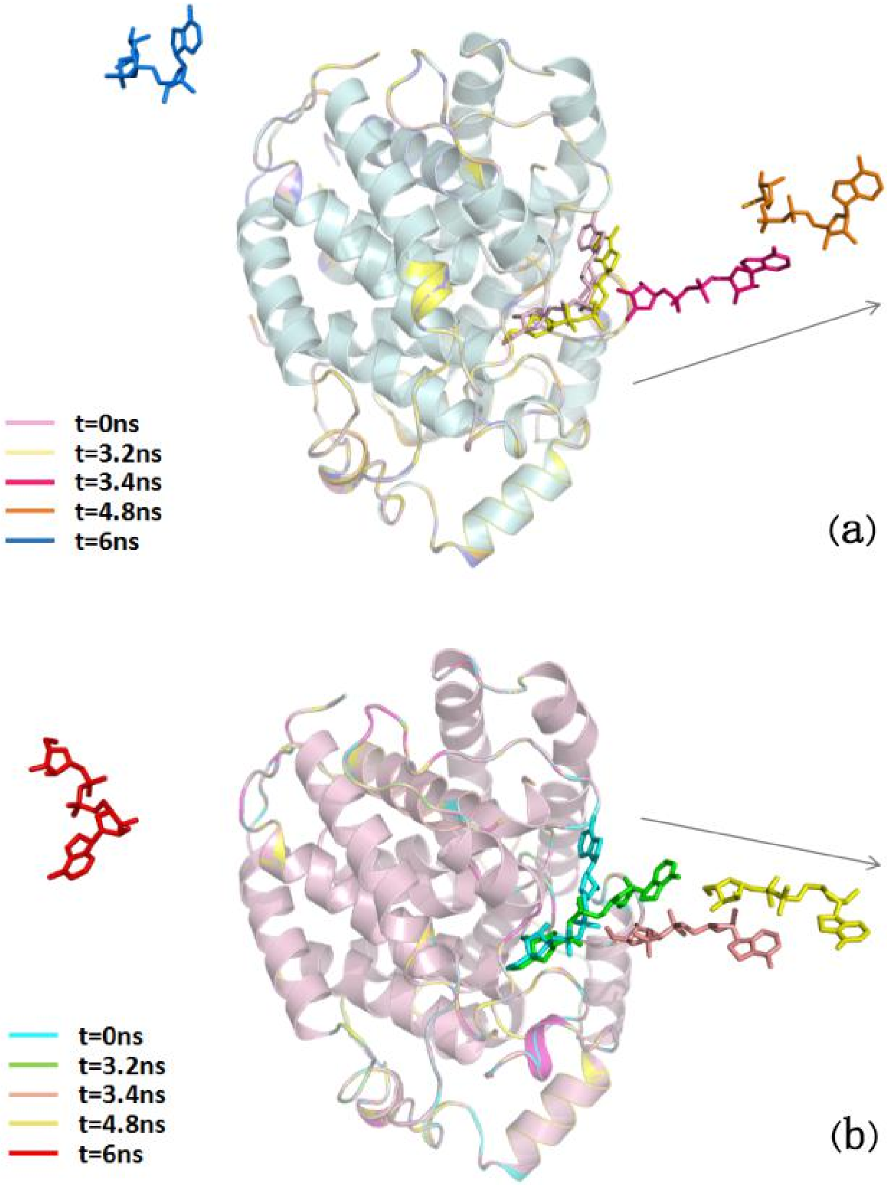

Table S1 Tunnel-lining residues for the non-phosphorylated type.

Residue index in PDB index	Amino Acid	Tunnel-lining Atoms
19	Tyr	CD1 CE1 HE1 CZ OH HH CE2 HE2
25	Glu	CB HB1 CG HG1 HG2 CD OE1 OE2
54	Ser	CB HB1 OG
55	Asp	HN CB HB1 HB2 CG OD1 OD2
56	Asp	HN CG OD1 OD2
98	Ala	CA CB HB2 HB3 C O
99	Pro	N CA HA CB C O
100	Gly	N HN CA HA1 HA2 C O
101	Gly	N HN
102	Ala	HN
103	Ser	HN CB HB1 HB2 HG1
124	Ser	N HN CA HA CB HB2 OG C O
264	Ser	N CA HA CB HB1 HB2 OG HG1 C O
269	Ser	CA HA CB HB1 HB2 OG HG1 C O
302	Asp	CG OD1 OD2
304	Asp	OD
97	Arg	O

Table S2 Tunnel-lining residues for the phosphorylated type.

Residue index in PDB index	Amino Acid	Tunnel-lining Atoms
19	TP	CA HA CB HB2 CG CD1 HD1 CE1 HE1 CZ CD2 HD2 CE2 HE2 OH P9 O10 O11 H11 O9
22	GLY	N CA HA1 HA2
25	GLU	CB HB1 CG HG2 CD OE1 OE2
54	SER	CA HA CB HB1 HB2 OG HG1 C
55	ASP	N HN CA CB HB2 CG OD1 OD2 C
56	ASP	N HN CB CG OD1 OD2
98	ALA	N CA HA CB HB1 HB2 HB3 C O
99	PRO	N CA HACB C O
100	GLY	N HN CA HA1 HA2
101	GLY	N HN
124	SER	N HN CA HA CB HB1 HB2 OG HG1 C O
126	GLU	CA HA CB HB2 CD OE2 C O
129	CYS	N HN
264	SER	N HN CA HA CB HB1 HB2 OG HG1 C O
269	SER	N CA HA CB HB1 HB2 OG HG1 C O
302	ASP	CA CB HB1 HB2 CG OD1 OD2
304	ASP	CB HB1 HB2 CG OD1 OD2
305	SER	OG
21	ASN	C
97	ARG	CA C O

Table S3 Hydrogen bond occupancies with non-phosphorylated ARH1 during MD simulations.

Residues	Non-phosphorylated ARH1	Occupancy(%)
**TYR4**	TYR4:HH...O:LEU140	81.84
**TYR19**	TYR19:HN...O:ASP15	81.24
	TYR19:O...HN:GLY22	53.28
	TYR19:O...OE1:GLU25	<5
	TYR19:O...OE2:GLU25	<5
**TYR20**	TYR20:HN...O:ALA16	8.04
	TYR20:O...HN:LYS23	32.08
**TYR205**	TYR205:HH...OE2:GLU157	13.28
	TYR205:HH...OE1:GLU157	78.04
	TYR205:HN...O:GLU201	9.92
	TYR205:HN...O:ALA202	<5
	TYR205:O...HE22:GLN208	<5
	TYR205:O...HN:GLN208	<5
	TYR205:O...HN:SER209	82.36
	TYR205:O...HB:SER209	8.80

Table S4 Hydrogen bond occupancies with phosphorylated ARH1 during MD simulations.

Residues	Phosphorylated ARH1	Occupancy(%)
**TYR4**	TP4:O...HN:MET8	<5
**TYR19**	TP19:HN...O:ASP15	67.16
	TP19:O...HN:GLY22	33.68
	TP19:H9...O:GLY96	30.84
	TP19:H10...O:GLY96	44.12
	TP19:OH...HA:ARG97	<5
**TYR20**	TP20:HN...O:ALA16	44.60
	TP20:O...HN:LYS23	49.12
**TYR205**	TP205:O9...HH22:ARG119	<5
	TP205:H10...OE2:GLU157	40.24
	TP205:H10...OE1:GLU157	19.44
	TP205:H9...OE2:GLU157	23.00
	TP205:H9...OE1:GLU157	15.44
	TP205:HN...O:GLU201	27.56
	TP205:HN...O:ALA202	7.44
	TP205:O...HE21:GLN208	<5
	TP205:O...HN:GLN208	<5
	TP205:O...HN:SER209	66.48
	TP205:O...HB:SER209	<5
	TP205:O...HG:SER209	59.76
