# Supplementary figures and images for: Understanding the differences of the ligand binding/unbinding pathways between phosphorylated and non-phosphorylated ARH1 using molecular dynamics simulations
Source: Sci Rep. 2017 Sep 29;7:12439. doi: 10.1038/s41598-017-12031-0 (PMC5622063; doi:10.1038/s41598-017-12031-0)

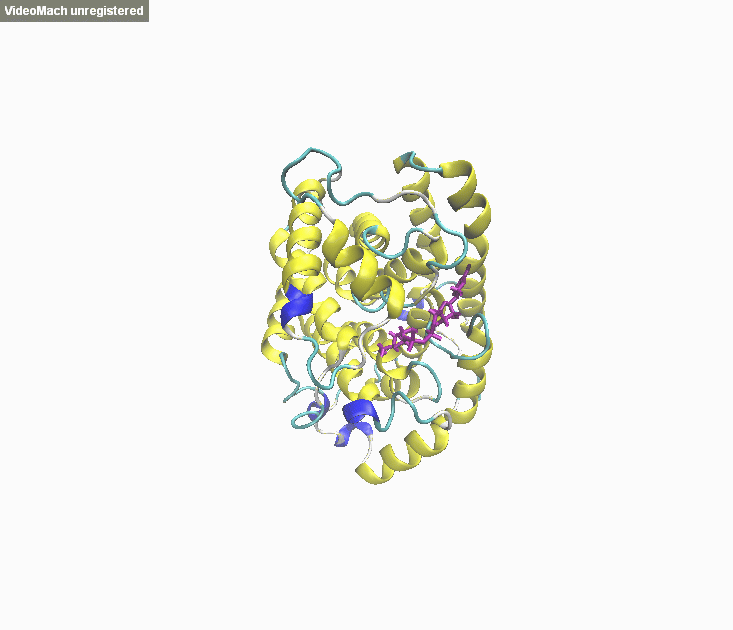

Supplement: Supplementary file 2 — The trajectory of SMD simulation for the non-phosphorylated [file 41598_2017_12031_MOESM2_ESM.gif]

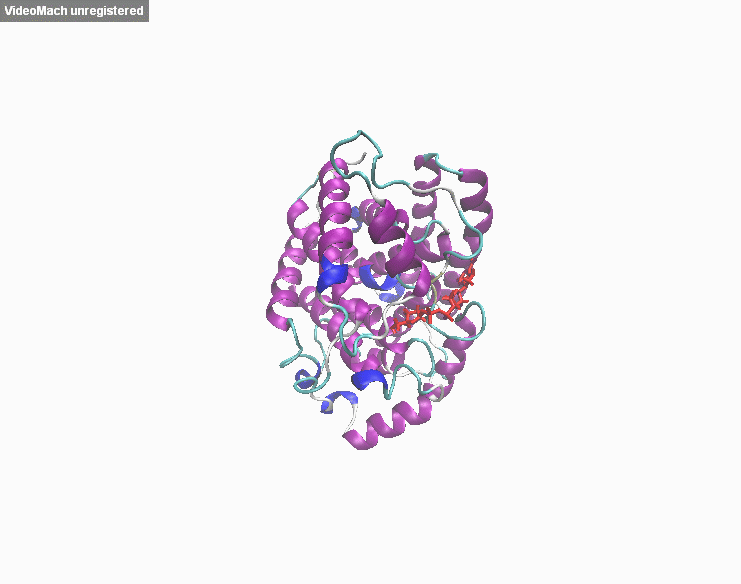

Supplement: Supplementary file 3 — The trajectory of SMD simulation for the phosphorylated [file 41598_2017_12031_MOESM3_ESM.gif]
